# *XXYLT1* and Mendelian Retinal Dystrophy

**DOI:** 10.1001/jamaophthalmol.2026.2795

**Published:** 2026-07-30

**Authors:** Minna Kraatari-Tiri, Hina Ishtiaq, Jaakko Tyrmi, Siying Lin, Andriana Valkama, Timo Tiirikka, Sara Äikäs, Laura Lähteenoja, Genevieve Wright, Elena Schiff, Aleksandr Jestin, Andrew R. Webster, Omar A. Mahroo, Michel Michaelides, Jukka Moilanen, Katri Pylkäs, Johannes Kettunen, Gavin Arno, Aura Falck, Tuomo Mantere, Elisa Rahikkala

**Affiliations:** 1 Department of Clinical Genetics, Oulu University Hospital, Oulu, Finland; 2 Research Unit of Clinical Medicine and Medical Research Center Oulu, Oulu University Hospital and University of Oulu, Oulu, Finland; 3 Center for Life Course Health Research, Faculty of Medicine, University of Oulu, Oulu, Finland; 4 Medical Research Center Oulu, Oulu University Hospital and University of Oulu, Oulu, Finland; 5 Division of Evolution, Infection and Genomics, School of Biological Sciences, Faculty of Biology, Medicine and Health, University of Manchester, Manchester, United Kingdom; 6 Manchester Centre for Genomic Medicine, Saint Mary’s Hospital, Manchester University NHS Foundation Trust, Manchester Academic Health Science Centre, Manchester, United Kingdom; 7 National Institute of Health Research Biomedical Research Centre at Moorfields Eye Hospital, London, United Kingdom; 8 University College London Institute of Ophthalmology, University College London, London, United Kingdom; 9 Northern Finland Laboratory Centre Nordlab, Oulu, Finland; 10 Department of Ophthalmology, Oulu University Hospital, Oulu, Finland; 11 Section of Ophthalmology, King’s College London, St Thomas’ Hospital Campus, London, United Kingdom; 12 Biocenter Oulu, University of Oulu, Oulu, Finland; 13 Laboratory of Cancer Genetics and Tumor Biology, Translational Medicine Research Unit, University of Oulu, Oulu, Finland; 14 Greenwood Genetic Center, Greenwood, South Carolina; 15 Department of Genomics, Laboratory Division, Turku University Hospital, Turku, Finland

## Abstract

**Importance:**

Substantial unexplained heritability remains for pathogenic inherited retinal disease (IRD) variants. Application of genome-wide association studies (GWAS) could help identify causal genes in rare diseases.

**Objective:**

To leverage a GWAS for the discovery of IRD-associated genes.

**Design, Setting, and Participants:**

This GWAS analysis was combined with replication of findings in 2 independent IRD cohorts. The study was conducted from January 2024 to December 2025 in a multicenter setting through FinnGen, 100 000 Genomes Project, and the National Health Service Genomic Medicine Service combined with clinical cohort from the Oulu University Hospital. Using IRD criteria from the *International Classification of Diseases*, *9th and 10th Revisions*, 540 individuals with IRD and 473 945 control individuals were identified in the FinnGen study. For validation of FinnGen results, 49 patients were recruited from Oulu University Hospital. Results were further validated in 2 individuals identified from the UK cohort.

**Main Outcomes and Measures:**

The GWAS and proteomics analysis were performed in the FinnGen cohort. Sanger and whole-genome sequencing and RNA approaches were used in a clinical IRD cohort to validate pathogenicity of the identified *XXYLT1* variant.

**Results:**

This GWAS identified 13 recessive loci reaching genome-wide significance (defined as *P* < 5 × 10^−8^). Of these, 4 (near or within *XXYLT1, ANKRD10, DYM*, and *CBLN4*) had not been associated with IRD, including the *XXYLT1* c.505-1G>C founder variant. This variant was further genotyped in the clinical replication cohort, leading to identification of 5 more homozygous individuals from 4 families. The phenotype was consistent with a cone-rod or macular dystrophy, with visual deterioration, cystoid macular edema and/or schisislike macular abnormalities. The effect of the *XXYLT1* c.505-1G>C variant was further investigated using RNA sequencing and complementary DNA amplicon sequencing, demonstrating exon 2 skipping and a loss-of-function effect. These findings were replicated in an independent population identifying 2 patients from the UK harboring a homozygous *XXYLT1* c.766G>A, p.(Glu256Lys) missense variant.

**Conclusions and Relevance:**

This GWAS identified an association between *XXYLT1* and IRD. These results affirm that GWAS in a founder population can be used as a potential tool for the discovery of rare mendelian disease genes and that *XXYLT1* should be considered in clinical IRD gene panels.

## Key Points

**Question:**

Can a genome-wide association study (GWAS) approach be used to identify genes associated with inherited retinal disease (IRD)?

**Findings:**

Using a recessive model, this GWAS identified 13 loci (9 known and 4 previously unknown putative loci) with genome-wide significance. One of the identified genes, *XXYLT1*, was confirmed as a rare mendelian IRD gene in independent Finnish and UK clinical cohorts; an *XXYLT1* c.505-1G>C founder variant showed a loss-of-function effect.

**Meaning:**

These findings support the need to include *XXYLT1* in clinical IRD gene panels.

## Teaser

This genome-wide association study evaluates genes associated with retinal dystrophy.

## Introduction

Inherited retinal diseases (IRDs) are a diverse group of rare, monogenic retinal disorders. They are typically characterized by progressive retinal impairment, which leads to gradual deterioration of visual function or complete blindness. IRDs are a leading cause of blindness among individuals aged 20 to 64 years and represent the most common cause of blindness in the working population,^1,2^ primarily due to progressive and irreversible photoreceptor degeneration. IRDs can be associated with other ocular complications, such as cataract, glaucoma, cystoid macular edema, and epiretinal membranes, which further impair vision.^3^ Although each IRD is individually rare, their collective incidence is estimated at 1 in 2000 to 1 in 4000 individuals.^4,5,6^

The etiology of IRDs is highly heterogeneous, and to date, 492 genes and loci have been implicated in the pathogenesis of IRDs.^7^ Next-generation sequencing is commonly used to identify pathogenic variants causing an IRD with a 52% to 74% diagnostic yield,^8^ although a substantial portion of heritability remains unexplained.

Genome-wide association studies (GWASs) have proven to be a powerful tool for uncovering genetic risk factors for multifactorial diseases. GWAS approaches have since been applied to complex ophthalmic diseases and traits, such as the thickness of the retinal nerve fiber layer^9^ and central serous chorioretinopathy.^10^ Medical literature contains limited research exploring the use of GWASs in monogenic diseases, and to our knowledge, no previous studies have sought to identify IRD-associated loci using this approach. Using FinnGen data, we discovered IRD-associated loci and confirmed *XXYLT1* as an IRD-associated gene in Finnish and UK cohorts that acts via a loss-of-function mechanism.

## Methods

### Ethics

This study took place from January 2024 to December 2025. All participants in the FinnGen study provided verbal or written informed consent for biobank research in accordance with the Finnish Biobank Act. The FinnGen study protocol was approved by the Coordinating Ethics Committee of the Hospital District of Helsinki and Uusimaa. The Oulu University Hospital IRD Cohort study was approved by the Northern Ostrobothnia Hospital District. The English cohort study was approved by the ethical review committee of Moorfields Eye Hospital and the Northwest London Research Ethics Committee. All the participants signed written informed consent prior to participation in the research project. Participants did not receive financial compensation. All research was conducted in accordance with the Declaration of Helsinki and relevant national legislation. Further details on FinnGen ethical approvals are available in eAppendix 2 in Supplement 1.

### FinnGen Project

Individuals diagnosed with an IRD (n = 540) and control individuals (n = 473 945) were identified from the FinnGen project (Data Freeze 12) using diagnosis codes 3627 and H35.5 from versions 9 and 10 of the *International Classification of Diseases* (*ICD-9* and *ICD-10*). To ensure accurate control selection, individuals with *ICD-10* codes H30-H36 were excluded. Further details on FinnGen protocols are available in the eMethods in Supplement 1. Clinical characteristics of both cases and controls are summarized in eTable 1 in Supplement 1. A flowchart of the study design is presented in eFigure 1 in Supplement 1. This report adhered to the Strengthening the Reporting of Observational Studies in Epidemiology (STROBE) reporting guideline and the Strengthening the Reporting of Genetic Association Studies (STREGA) reporting guideline extension for genetic association studies.

### Oulu University Hospital IRD Cohort

A total of 49 patients from Northern Finland diagnosed with an IRD of unknown genetic etiology (*ICD-10* code H35.5) were recruited at Oulu University Hospital, Oulu, Finland. All participants received comprehensive ophthalmological evaluations, and prior genetic testing results were negative (further details in the eMethods in Supplement 1).

### UK IRD Cohort

**Figure 1.**
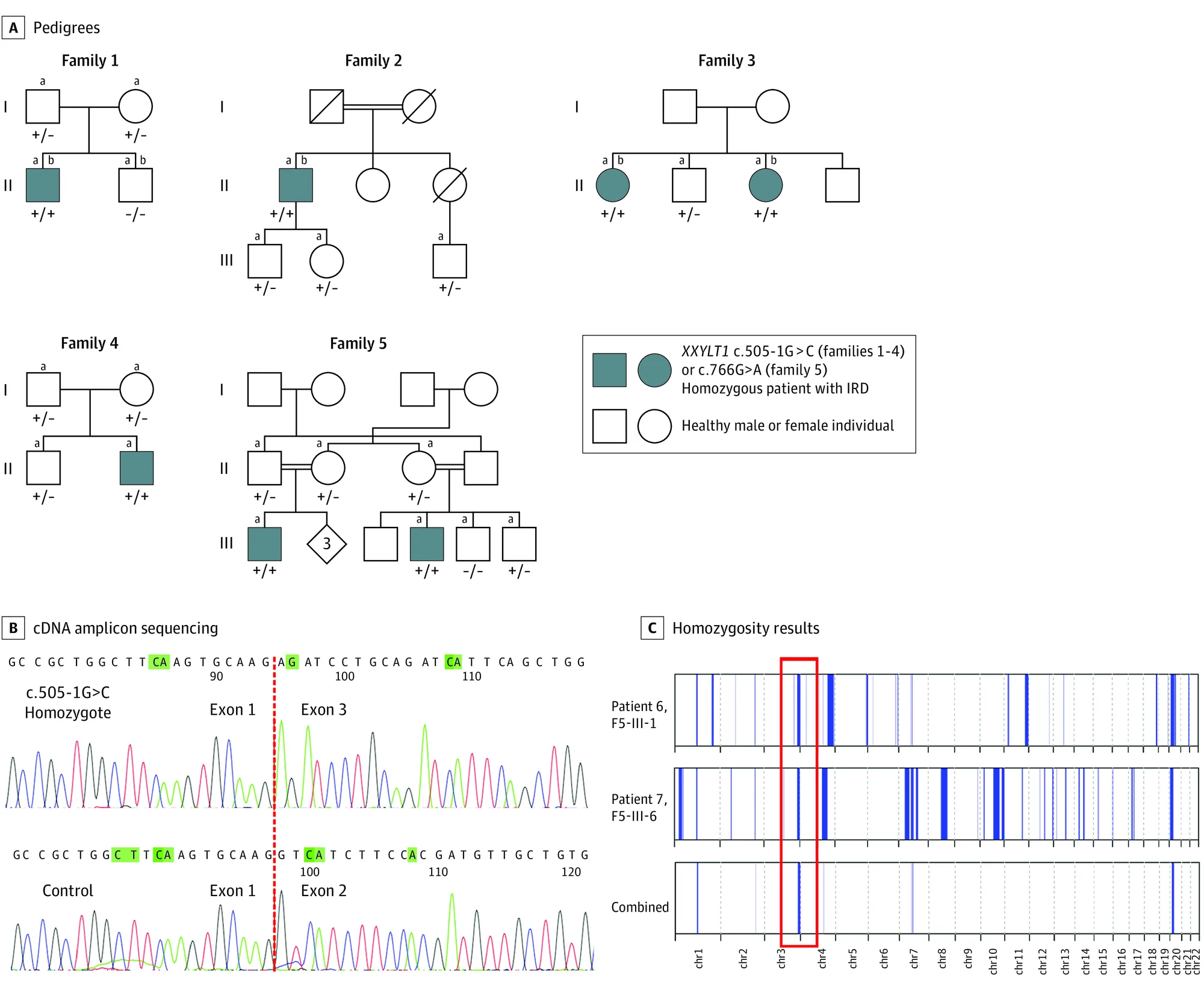
Molecular Investigations A, Pedigrees of patients homozygous for *XXYLT1* c.505-1G>C, p.? (families 1-4) and *XXYLT1* c.766G>A, p.(Glu256Lys) (family 5), demonstrating autosomal recessive segregation of variants. +/+ indicates homozygous; +/−, heterozygous; −/−, wild type. B, Complementary DNA (cDNA) amplicon sequencing using RNA extracted from cultured fibroblasts of a patient homozygous for *XXYLT1* c.505-1G>C revealed exon 2 skipping compared with a wild-type healthy control individual. C, Homozygosity results; regions of homozygosity are shown as vertical bars. The largest shared region of homozygosity between patient 6 and patient 7 (highlighted by the box outline) was an 11.21 Mb segment on chromosome 3 (chr3:g.184503000-195710647) encompassing the *XXYLT1* gene. IRD, indicates inherited retinal dystrophy. ^a^DNA available. ^b^Fibroblasts available.

Two male individuals, aged 29 and 30 years, from the same extended family were recruited via the Inherited Retinal Disease service at Moorfields Eye Hospital, London, UK. Both were diagnosed with an IRD of unknown genetic etiology (*ICD-10* code H35.5). Comprehensive assessments included detailed clinical, medical, and family histories, as well as in-depth ocular phenotyping (further details in the eMethods in Supplement 1). Standard genetic testing was negative. Due to the consanguineous and extended familial structure of family 5 (Figure 1), homozygosity mapping^11^ and subsequent research analysis of whole-genome sequencing (WGS) data were conducted. WGS was performed through the 100 000 Genomes Project (patient 6) and the National Health Service Genomic Medicine Service (patient 7)^12^ (Figure 1; Table 1; eTable 2 in Supplement 1).

**Table 1.** Clinical Characterization of Patients With Biallelic *XXYLT1* Variants^a^

Characteristic	P1 (F1-II-1)	P2 (F2-II-1)	P3 (F3-II-1)	P4 (F3-II-3)	P5 (F4-II-2)	P6 (F5-III-1)	P7 (F5-III-6)
Sex/age^b^	M/46 y	M/75 y	F/31 y	F/26 y	M/51 y	M/30 y	M/30 y
*XXYLT1* variant	c.505-1G>C	c.505-1G>C	c.505-1G>C	c.505-1G>C	c.505-1G>C	c.766G>A	c.766G>A
Age at first eye examination	35 y	50 y	6 y	8 y	>40 y	14 y	27 y
First symptoms and findings	Deterioration of vision, grainy pigmentation in the macula	Deterioration of vision, CMO	Compromised VA, grainy pigmentation in the macula	Glasses from second grade, night blindness, deterioration of VA, CMO	Deterioration of VA, macular edema in LE, GA in the maculae	Poor central vision since childhood	Gradual deterioration in central vision
Clinical phenotype	Cone-rod dystrophy	Cone-rod dystrophy	Early cone-rod dystrophy	Cone-rod dystrophy	Cone-rod dystrophy	Macular dystrophy	Macular dystrophy
BCVA, logMAR (Snellen)	1.30 (20/400)/1.20 (20/320)	0.20 (20/32)/0.20 (20/32)	0.10 (16/20)/0.10 (16/20)	1.20 (20/320)/0.90 (20/160)	0.40 (20/50)/0.20 (20/32)	1.00 (20/200)/0.80 (20/125)	0.80 (20/125)/1.00 (20/200)
Fundus photography and OCT	Grainy central pigmentation, later atrophy, waxy discs, OCT: damaged neuroepithelium	Pigmentations and atrophy in the central and peripheral retina, arterial narrowing, OCT: CMO, puckering	Schisislike change in the macular area and single cysts, grainy macula	CMO and foveal schisislike change; glaucomatous disc RE	CMO in LE, papillomacular atrophy in both eyes	Foveal schisis, ORD involving the macula, central macular pigmentation	ORD involving the macula, no macular schisis, central macular pigmentation
ERG and VEP	ERG: low amplitudes	ERG: low amplitudes	ERG: interpreted normal at 16 y	ERG: low amplitudes, especially scotopic	ERG: low amplitudes	ERG: macular dysfunction, mild generalized retinal dysfunction	ERG: macular dysfunction, mild generalized retinal dysfunction
	VEP: normal	VEP: slightly delayed latencies	VEP: normal	VEP: normal	VEP: delayed latencies	NA	NA
Visual field	Bilateral ring scotoma	Bilateral ring scotoma	Normal	Low sensitivity peripheral narrowing	Bitemporal scotoma in the central field	NA	NA
Cataract	Yes, mild PSC	Yes, NS, operated	No	Yes, operated, PCO	No	No	No
Additional ocular symptoms	Night blindness	Night blindness, glare	Glare, progressive night vision problems, symptoms of dry eye, exophoria	Intermediate uveitis, secondary glaucoma in both eyes, night blindness, glare	Night blindness	No	No

Abbreviations: BCVA, best-corrected visual acuity; CMO, cystoid macular edema; ERG, electroretinogram; F, female; GA, geographic atrophy; LE, left eye; M, male; NA, not applicable; NS, nuclear sclerosis; OCT, optical coherence tomography; ORD, outer retinal disruption; PCO, posterior capsule opacification; PSC, posterior subcapsular cataract; RE, right eye; VA, visual acuity; VEP, visual evoked potential.

^a^ More detailed table in eTable 2 in Supplement 1.

^b^ Age at the time of the study.

### Genotyping and Genotype Imputation of Variants

Genotyping of FinnGen samples was performed using Illumina and Affymetrix arrays (Illumina Inc and Thermo Fisher Scientific, respectively). Genotype imputation was conducted using the SISu version 3 reference panel, which contains 3775 whole genomes (https://www.protocols.io/view/genotype-imputation-workflow-v3-0-xbgfijw).

### Genome-Wide Association Analysis

GWAS analyses were performed using REGENIE software version 3.3,^13^ applying both additive and recessive genetic models. Covariates included age, 10 first principal components of genetic ancestry, and genotyping batch.

### Colocalization

Colocalization analyses were conducted using the software package coloc^14^ between retinal dystrophy association signals and protein quantitative trait loci identified in the FinnGen Somascan dataset. Protein quantitative trait loci are genomic regions where inherited variation influences the abundance of specific proteins. Colocalizations were considered significant if the posterior probability of a shared causal variant was 0.8 or greater.

### *XXYLT1* Genotyping and Whole-Genome Sequencing

A replication analysis of the GWAS identifying the *XXYLT1* c.505-1G>C variant was conducted using targeted Sanger sequencing in an IRD cohort at Oulu University Hospital (n = 49). Segregation analyses of the *XXYLT1* c.505-1G>C variant were performed in the families of 4 homozygous probands. WGS was performed for the sister of proband 3, who was also homozygous for the *XXYLT1* c.505-1G>C variant, to exclude other potential monogenic causes.

In addition, 41 Finnish patients with IRD of unknown etiology underwent genome sequencing. Lead variants identified in the FinnGen dataset were analyzed to assess replication and potential functional relevance. Linkage disequilibrium between the intergenic FinnGen lead variants and known pathogenic IRD founder variants was also evaluated in the Oulu University Hospital IRD cohort by examining the co-occurrence of each lead single-nucleotide variant (SNV) with the corresponding nearby pathogenic IRD gene variant.

### Transcriptomic Analysis and Complementary DNA Amplicon Sequencing

Total RNA was extracted from skin fibroblasts obtained from 4 affected individuals homozygous for the *XXYLT1* c.505-1G>C variant and 2 control individuals with wild-type alleles. Messenger RNA (mRNA) sequencing was used to quantify *XXYLT1* expression and perform differential gene expression analysis. In addition, the splicing effects of the *XXYLT1* c.505-1G>C variant were investigated using complementary DNA amplicon sequencing. Details regarding fine mapping of GWAS loci, annotation of loci with biological function, and detailed molecular protocols are provided in the eMethods in Supplement 1.

## Results

### Genome-Wide Association Study in FinnGen

**Figure 2.**
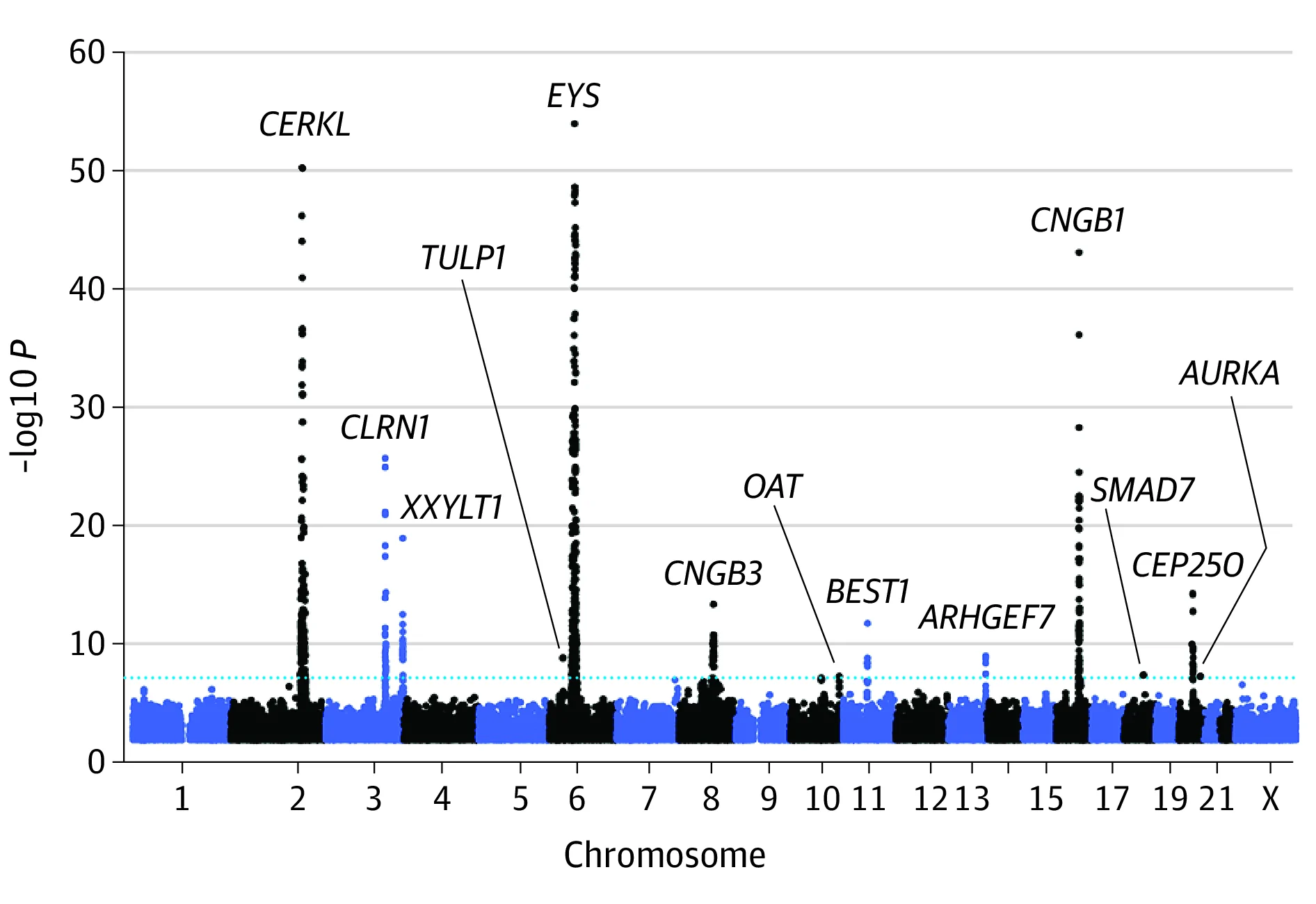
Manhattan Plot of the Recessive Case-Control Genome-Wide Association Study The FinnGen cohort comprised 540 individuals with inherited retinal dystrophy and 473 945 control individuals. Chromosomal positions are displayed on the x-axis and –log10 (*P* value) is plotted on the y-axis for each single-nucleotide variant. Thirteen loci reached genome-wide significance (*P* < 5 × 10^−8^). The candidate gene associated with each locus is labeled above the corresponding peak.

Using a recessive model, this GWAS identified 13 loci reaching genome-wide significance (*P* < 5 × 10^−8^) (Figure 2; Table 2^15,16,17,18,19,20,21,22,23,24,25,26,27^; eTable 3 in Supplement 1). Of these, 9 contained genes previously shown to be causative for mendelian IRD, supporting the validity of a GWAS in identifying known monogenic disease genes within a Finnish founder population. Four of the 13 loci had no known IRD genes located within a 2-Mb window of the lead variants. Lead variants for each locus are detailed in Table 2. To the authors’ knowledge, none of these loci have been previously associated with an IRD in a GWAS. Detailed analyses of the lead variants are provided in the eResults in Supplement 1.

**Table 2.** Lead Single-Nucleotide Variants From a Genome-Wide Association Study With a Recessive Model

Lead variant (GRCh38)^a^	Lead variant rsID	EAF	*P* value	Homozygotes in cases/controls, No. (%)^b^	Nearest gene^c^	Type	Candidate gene^d^	Pheno-type MIM No.	ClinVar variation ID	Current status of the variant	Source
2:181603943:G>C	rs200711686	0.00555	5.8 × 10^−51^	20 (3.7)/<5 (<0.001)	*CERKL*	Missense	*CERKL*	608380	866659	Known pathogenic	Tuson et al^15^
3:150902877:G>A	rs148594758	0.00499	1.7 × 10^−26^	11 (2.0)/<5 (<0.001)	*CLRN1*	Intronic	*CLRN1*	614180, 276902	NA	Nearby known pathogenic	Sankila et al^16^; Khan et al^17^
3:195226857:C>G	rs201922399	0.00507	9.7 × 10^−20^	7 (1.3)/5 (0.001)	*XXYLT1*	Splice acceptor	*XXYLT1* ^f^	NA	NA	Novel candidate^e^	Present study
6:34017689:C>T	rs1247877008	0.00173	1.1 × 10^−9^	<5 (<0.9)/0	*GRM4*	Downstream gene	*TULP1*	600132	NA	Nearby known pathogenic	Hagstrom et al^18^
6:63721375:TTCTGCATG>T	rs528919874	0.00714	1.1 × 10^−54^	22 (4.1)/9 (0.002)	*EYS*	Frameshift	*EYS*	602772	550019	Known pathogenic	Abd El-Aziz et al^19^
8:86643780:AG>A	rs397515360	0.00271	3.6 × 10^−14^	5 (0.9)/<5 (<0.9)	*CNGB3*	Frameshift	*CNGB3*	262300	5225	Known pathogenic	Sundin et al^20^
10:124144264:AGTGT>A	rs1282805098	0.00264	4.1 × 10^−8^	<5 (<0.9)/<5 (<0.001)	*CHST15*	Intergenic	*OAT*	258870	NA	Nearby known pathogenic	Mitchell et al^21^
11:61955892:G>A	rs121918284	0.00202	1.5 × 10^−12^	<5 (<0.9)/<5 (<0.001)	*BEST1*	Missense	*BEST1*	193220, 611809, 153700, 613194	2740	Known pathogenic	Graff et al^22^; Burgess et al^23^; Yardley et al^24^; Davidson et al^25^
13:110914511:G>A	rs1020045634	0.000764	8.8 × 10^−10^	<5 (<0.9)/<5 (<0.001)	*ANKRD10*	Noncoding transcript exon variant	*ARHGEF7* ^f^	NA	NA	Novel candidate^e^	Present study
16:57901371:T>A	rs201162411	0.00625	7.9 × 10^−44^	18 (3.3)/14 (0.003)	*CNGB1*	Missense	*CNGB1*	613767	166891	Known pathogenic	Bareil et al^26^
18:49086805:T>C	rs529344081	0.00205	3.2 × 10^−8^	<5 (<0.9)/<5 (<0.001)	*DYM*	Intronic	*SMAD7* ^f^	619868, 251850	NA	(This study)^e^	NA
20:35292551:G>A	rs771075130	0.00317	4.3 × 10^−15^	5 (0.9)/<5 (<0.001)	*FAM83C*	Upstream gene	*CEP250*	618358	NA	Nearby known pathogenic	Khateb et al^27^
20:55873222:T>C	rs186466996	0.000588	4.2 × 10^−8^	<5 (<0.9)/0	*CBLN4*	Intergenic	*AURKA* ^f^	NA	NA	(This study)^e^	NA

Abbreviations: EAF, effect allele frequency; NA, not available.

^a^ Lead variant, chromosome, base-pair position, reference allele and effect allele (formatted as “chromosome:position:reference>effect”).

^b^ Number of homozygotes in cases (% of all cases)/controls (% of all controls), the count and proportion of homozygous individuals among IRD cases (n = 540) and controls (n = 473 945).

^c^ Nearest gene is the closest protein coding gene to the lead variant.

^d^ Candidate gene refers to the gene presumed to drive the association signal based on functional evidence and prior literature.

^e^ This study identified no effect allele for variants in independent cohorts except XXYLT1.

^f^ Previously unknown recessive loci.

### Description of Recessive Loci in FinnGen

We identified 4 previously unknown recessive loci (Figure 2; Table 2). The recessive lead variants of these loci were rs201922399 (GRCh38 chr3-195226857-C>G) located in intron 1 of the *XXYLT1* gene, rs1020045634 (GRCh38 13-110914511-G>A) within a noncoding transcript of *ANKRD10*, rs529344081 (GRCh38 18-49086805-T>C) located in an intron of *DYM* and rs186466996 (GRCh38 20-55873222-T>C) near *CBLN4*. Beyond the *XXYLT1* locus, fewer than 5 individuals in FinnGen were homozygous for the lead variant.

### A GWAS With an Additive Model

A GWAS using an additive model identified 7 loci with genome-wide significance (*P* < 5 × 10^−8^) (eFigure 2 and eTable 4 in Supplement 1). Of these, 6 loci contained genes previously associated with an IRD. A previously unknown locus was identified, with lead variant rs150413925 (GRCh38 3-83682513-T>C) located near *CADM2*.

None of the previously unknown lead variants (rs1020045634, rs529344081, rs186466996, and rs150413925) were detected in patients from the Oulu University Hospital IRD cohort who underwent WGS (n = 42). A more detailed description of these loci is provided in the eResults in Supplement 1.

### Description of *XXYLT1* Loci as IRD Associated

The lead variant at chromosome 3 locus (rs201922399) is a splice acceptor variant located in intron 1 of the *XXYLT1* gene (NM_152531.5 c.505-1G>C, p.? GRCh38 chr3-195226857C>G). *XXYLT1* has not previously been associated with an IRD or other human diseases. This variant is enriched in the Finnish population, with an allele frequency of 0.006299 in gnomAD version 4.1.0 compared with 0.00003306 gnomAD version 4.1.0 compared with 0.00003306 in non-Finnish European individuals (191-fold enrichment), and the Finnish dataset includes 1 homozygous individual. Based on population frequency, the expected number of *XXYLT1* c.505-1G>C homozygous individuals in Finland is approximately 0.006299^2^ × 5.5 million = 218. According to SpliceAI,^28^ the variant is predicted to result in loss of the canonical intron 1 acceptor site, possibly leading to exon 2 skipping or, alternatively, an in-frame deletion of 12 amino acid residues via an alternative acceptor site within exon 2.

In the FinnGen project, 19 individuals were homozygous and 5069 were heterozygous for the *XXYLT1* c.505-1G>C variant, out of a total of 520 210 participants, corresponding to an allele frequency of 0.00491. Among the homozygous individuals, 10 (53%) had a preexisting diagnosis of an IRD or macular dystrophy with a median (range) age of 38 (13-76) years at diagnosis. The remaining homozygous individuals showed various other eye-related conditions (eTable 5 in Supplement 1). The *XXYLT1* c.505-1G>C variant also showed significant associations in the FinnGen dataset in additional GWAS analyses of ophthalmic end points, including retinal disorders; disorders of the choroid and retina; macular and posterior pole degeneration; degenerative macular disease; other specified or unspecified retinal disorders; and visual impairment, including binocular or monocular blindness.

### Colocalization Analysis

Colocalization analysis between retinal dystrophy association signals and protein quantitative trait loci from the FinnGen Somascan dataset identified 34 genomic regions with a posterior probability of 0.8 or greater for a shared causal variant. The strongest colocalization signal of all recessive signals was observed between the *XXYLT1* association locus and XXYLT1 protein levels, with the effect allele G associated with reduced protein expression (β, –2.2; 95% CI, −2.73 to −1.63; *P* = 2.6 × 10^−14^).

### Replication of the *XXYLT1* Results

To investigate further the association between the *XXYLT1* c.505-1G>C variant and the mendelian IRD, targeted Sanger sequencing was performed in the molecularly unsolved IRD cohort at Oulu University Hospital (eFigure 3 in Supplement 1). Five patients from 4 distinct families were found to be homozygous for the variant (Figure 1; Table 1), and the variant segregated in an autosomal recessive manner in the respective families (Figure 1). WGS was performed for 1 homozygous patient (patient 4, F3-II-3 in Figure 1), excluding other known genetic causes of IRDs.

Through the European Retinal Disease Consortium (ERDC) network, 2 additional IRD patients from a large consanguineous family (Figure 1A, Pedigree 5) were identified as homozygous for another *XXYLT1* variant. Homozygosity mapping and subsequent WGS analysis of shared regions of homozygosity revealed a homozygous *XXYLT1* c.766G>A, p.(Glu256Lys) variant (GRCh38:chr3:195156468C>T) in both affected individuals. The variant resides within the largest shared region of homozygosity, an 11.21 Mb interval on chromosome 3 (chr3:g.184503000-195710647; Figure 1C). No other plausible candidate variants were identified within this interval. The *XXYLT1* c.766G>A variant affects highly conserved glutamine residue at position 256 (p.Glu256). Computational predictions consistently support a deleterious impact: Combined Annotation Dependent Depletion score of 26.8, Rare Exome Variant Ensemble Learner score of 0.567, PolyPhen2 score of 1.0 (probably damaging), Sorting Intolerant From Tolerant score of 0.03 (deleterious), MutationTaster (deleterious), and AlphaMissense score of 0.987 (deleterious). Segregation analysis confirmed an autosomal recessive inheritance pattern in the family.

### Clinical Characteristics of *XXYLT1* Homozygous Patients

**Figure 3.**
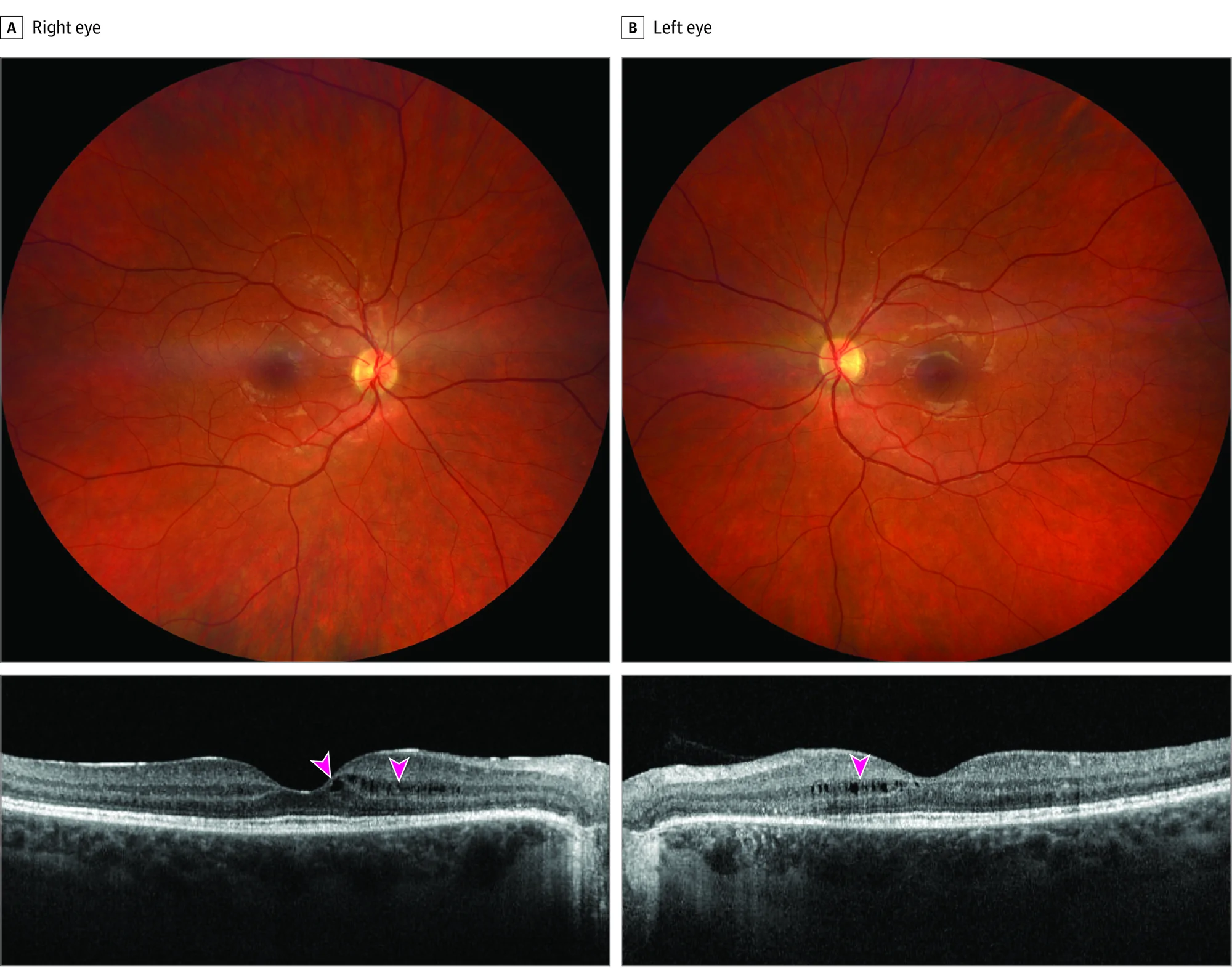
Ophthalmological Findings Retinal imaging of patient 3, including fundus (top; Zeiss Clarus 700) and macular optical coherence tomography (bottom; OCT) (Topcon Corporation) scans. OCT images demonstrate schisislike changes in the macular region (right and left eye arrowheads) as well as a single, slightly larger, cystlike dark cavity (right eye arrowhead), which are typical early lesions in *XXYLT1*-related inherited retinal dystrophy.

Patients with biallelic *XXYLT1* c.505-1G>C splice-site variants exhibited a typical cone–rod dystrophy phenotype, whereas those with biallelic *XXYLT1* c.766G>A, p.(Glu256Lys) missense variants showed macular dystrophy. Deterioration of vision was the initial symptom in all 7 cases from 5 families (Table 1; Figure 3; eTable 2 and eFigure 4 in Supplement 1). Initial ophthalmological examinations occurred at a mean (range) age of 27 (6-50) years. Visual impairment was classified as severe in 3 individuals, moderate in 1, mild in 2, and absent in 1. Early ophthalmological findings included macular lesions, cystoid macular edema, or pigmentary changes in the macula and difficulty seeing in low-light conditions. Retinal changes featured schisislike changes in the foveal region in at least 3 individuals, which appeared to progress to macular atrophy. These changes corresponded with central visual field defects. ERG revealed low amplitudes indicating rod and cone dysfunction in 6 of the 7 cases. Additionally, patient 1 had mild posterior subcapsular cataracts, patient 2 had nuclear cataracts, and patient 4 presented with posterior capsule opacification following cataract surgery, in the context of chronic uveitis and its treatment.

### RNA and Complementary DNA Sequencing Results

Based on mRNA sequencing results, *XXYLT1* expression in all 4 studied *XXYLT1* c.505-1G>C homozygous patients was markedly reduced (mean [range] transcripts per million, 2.7 [1.8-3.3]) compared with control individuals with wild-type alleles (mean [range] transcripts per million, 24.3 [24.1-24.6]). Manual inspection of residual reads from all patient samples using an integrative genomics viewer revealed consistent skipping of exon 2. Details of differentially expressed genes with a log_2_ fold-change ≥2.0 or ≤−2.0 and an adjusted *P* value ≤.001 are listed in eTable 6 in Supplement 1. The heatmap of the top 30 differentially expressed genes is presented in eFigure 5 in Supplement 1. Consistent with the mRNA sequencing findings, targeted complementary DNA amplicon sequencing of residual mRNA from homozygous patients confirmed exon 2 skipping, resulting in a premature stop codon (Figure 1B). These results suggest that the *XXYLT1* c.505-1G>C variant likely triggers nonsense-mediated decay, providing evidence for a splice defect leading to a loss-of-function effect.

## Discussion

In this GWAS analysis of the FinnGen project, 13 recessive and 7 additive loci were observed with genome-wide significance (*P* < 5 × 10^−8^), including 4 previously unknown loci, 1 of which, *XXYLT1*, was validated in an independent Finnish cohort of patients with IRD of previously unknown etiology. In the independent cohort, 10% (n = 5/49) of patients were homozygous for the *XXYLT1* c.505-1G>C splice acceptor variant segregating in an autosomal recessive manner across 4 families (Figure 1). RNA and complementary DNA sequencing of skin fibroblasts revealed markedly reduced *XXYLT1* expression in patients homozygous for the *XXYLT1* c.505-1G>C variant. This variant leads to exon 2 skipping and introduces a premature stop codon, providing strong evidence for a loss-of-function effect. Through international collaboration, 2 additional IRD patients homozygous for an *XXYLT1* c.766G>A, p.(Glu256Lys) missense variant were identified, further supporting *XXYLT1* as an IRD-associated gene.

The *XXYLT1* gene encodes xyloside xylosyltransferase 1, not previously linked to any human diseases. Homozygous *XXYLT1* knockout mice exhibited abnormal retinal morphology (http://www.mousephenotype.org),^29^ supporting its essential role in retinal function. These mice also exhibited abnormal vibrissa (whisker) morphology, decreased lean body mass, and increased total body fat. In our study, patients homozygous for the *XXYLT1* c.505-1G>C and c.766G>A variants presented with isolated forms of IRDs and normal lean body mass, suggesting tissue-specific effects.

XXYLT1 negatively regulates Notch signaling, a cell-to-cell communication pathway that plays a critical role in cell fate decisions, by modifying the Notch receptors through glycosylation.^30^ These glycans are essential for proper Notch signaling, and variants in the glycosyltransferases that synthesize O-glycans disrupt Notch activity and are linked to multiple congenital disorders.^31^ The role of the xylosyl extension in O-glucose glycans has been assessed by measuring cell-surface NOTCH1 and NOTCH2 levels in HEK293T cells lacking the relevant xylosyltransferase genes. These knockout cells showed impaired receptor trafficking. Consistent with this, loss of XXYLT1 resulted in incomplete O-glucose glycan elongation.^32^

Zebrafish models of an IRD have demonstrated that Notch inhibition promotes photoreceptor regeneration.^33^ Experimental findings have shown that interactions between pericytes and endothelial cells are essential for maintaining retinal vasculature stability and function,^34,35^ and interference with Notch signaling disrupts retinal cell differentiation, resulting in numerous variant clones with aberrant morphology.^36^

Differential gene expression analysis of skin fibroblasts from patients homozygous for *XXYLT1* c.505-1G>C and healthy control individuals revealed notable differences (eFigure 5 and eTable 6 in Supplement 1). *XXYLT1* expression was markedly reduced in all homozygous patients compared with control individuals. Among differentially expressed genes, *TNIK* (eTable 6 in Supplement 1) has been found to modulate Notch signaling, particularly in the context of Wnt-Notch pathway crosstalk, where it may influence transcriptional regulation downstream of Notch activation. It is a known effector in the Wnt/β-catenin pathway, acting as a transcriptional coactivator of TCF4 to promote the expression of Wnt target genes. Indirect interactions between Wnt and Notch signaling have been proposed, whereby *TNIK* may influence Notch target gene expression through shared transcriptional regulators, such as HES1 or MYC.^37^

Genetic variation in the *ELN* (elastin) gene, particularly rs2301995, has been associated with increased age-related macular degeneration risk in Japanese individuals, highlighting the role of elastin-related pathways in retinal disease.^38^ One of the highly differentially expressed genes was *ELN* (eTable 6 in Supplement 1), which encodes elastin. Degradation of elastin has been shown to impair retinal structure and function and has been associated with the development and progression of age-related macular degeneration, particularly its neovascular forms.^39,40^

*XXYLT1* variants were typically associated with schisislike changes in the fovea, macular lesions progressing to macular atrophy, and corresponding central visual field defects. However, considerable phenotypic variability was observed among affected individuals, even within the same families (Table 1; Figure 3). Additional research is required to characterize the full phenotypic spectrum associated with pathogenic *XXYLT1* variants.

Notably, 1 individual homozygous for the *XXYLT1* c.505-1G>C variant was identified in the gnomAD database, and 5 such individuals were found among the FinnGen control group. This may reflect the relatively late-onset clinical presentation of IRDs (median [range] age at diagnosis, 38 [13-76] years in FinnGen) associated with this variant.

FinnGen and other biobank initiatives in founder populations offer a powerful platform for identifying monogenic recessive disease genes beyond common SNV risk factors, through GWAS, owing to the enrichment of specific variants. In this study, we identified 8 loci harboring known monogenic IRD genes and a causal IRD gene, *XXYLT1,* demonstrating the utility of this approach for discovering new monogenic disease genes. Additionally, we identified 4 other loci: recessive rs1020045634 within *ANKRD10,* rs529344081 in the intron of *DYM*, rs186466996 near *CBLN4* (Table 2; eDiscussion in Supplement 1), and an additive rs150413925 near *CADM2* (eTable 4 in Supplement 1). Further investigation in additional whole-genome–sequenced IRD cohorts is needed to determine whether these association signals represent true causal relationships. We also identified genes known to cause recessive IRD, such as *CNGB1*, using an additive model in GWAS demonstrating the large effect size of the disease-associated homozygotes.

### Limitations

This study has limitations. Most FinnGen samples were genotyped using a customized array containing approximately 700 000 SNV markers, which may be suboptimal for detecting rare causative variants, as SNV arrays can be unreliable for genotyping very rare pathogenic variants.^41^ Despite this, we successfully replicated the association between an IRD and *XXYLT1* in 2 independent cohorts. However, the associations involving the other 3 recessive loci identified in this study could not be replicated in an independent Finnish IRD cohort. This may reflect false-positive associations or limited statistical power due to the small sample size of the clinical replication cohort.

This study investigated the *XXYLT1* gene association with mendelian IRD through a GWAS. Results of the study implicate GWASs as a potential tool to identify rare mendelian disease genes in founder populations. In complex disorders, GWASs and meta-analyses across diverse populations are typically used to validate associations. Meta-analysis was not conducted in this study, as IRDs are generally monogenic and often exhibit enrichment of population-specific variants,^42^ which complicates the reliable integration of findings across heterogeneous cohorts. Additionally, the results of the differential gene expression analysis should be interpreted with caution, as *XXYLT1* may exhibit tissue-specific expression patterns that are not reflected in skin fibroblasts.

## Conclusions

This GWAS identified an association between *XXYLT1* and IRDs, demonstrating that a GWAS approach in founder populations can enable the identification of monogenic, rare recessive candidate genes. *XXYLT1* was found to be an IRD-associated gene explaining a substantial portion of molecularly undiagnosed Finnish patients, with clinical phenotypes of either cone-rod dystrophy or macular dystrophy. To strengthen these discoveries, additional cohort-based studies are needed to validate the associations, alongside functional investigations to elucidate the mechanisms by which these candidate genes contribute to IRDs.

## Notes

Supplement 1. eMethods eResults eDiscussion eAppendix 1. Ethics declaration eAppendix 2. Acknowledgments eFigure 1. Flowchart of the study design eFigure 2. Manhattan plot of the additive case-control GWAS eFigure 3. Identification of rare XXYLT1 splice acceptor variant associated with IRD eFigure 4. Ophthalmological findings eFigure 5. Heatmap comparing gene expression profiles of XXYLT1 c.505-1G>C homozygous IRD patients and healthy controls, highlighting the top 30 differentially expressed genes (DEGs) eTable 1. Case and control characteristics of the FinnGen project eTable 2. Clinical characterization of patients with biallelic XXYLT1 variants eTable 3. Significant association of Recessive GWAS Intragenic lead SNPs with IRD eTable 4. Lead SNPs using an additive GWAS model eTable 5. Clinical characteristics of individuals homozygous for the XXYLT1 c.505-1G>C variant eTable 6. Summary of differentially expressed genes and their functions eReferences

Supplement 2. FinnGen Project members

Supplement 3. Data sharing statement
