# Supplementary material for: XXYLT1 and Mendelian Retinal Dystrophy
Source: JAMA Ophthalmol. 2026 Jul 30:e262795. Online ahead of print. doi: 10.1001/jamaophthalmol.2026.2795 (PMC13425242; doi:10.1001/jamaophthalmol.2026.2795)
Supplement: Supplement 1. — eMethods eResults eDiscussion eAppendix 1. Ethics declaration eAppendix 2. Acknowledgments eFigure 1. Flowchart of the study design eFigure 2. Manhattan plot of the additive case-control GWAS eFigure 3. Identification of rare XXYLT1 splice acceptor variant associated with IRD eFigure 4. Ophthalmological findings eFigure 5. Heatmap comparing gene expression profiles of XXYLT1 c.505-1G>C homozygous IRD patients and healthy controls, highlighting the top 30 differentially expressed genes (DEGs) eTable 1. Case and control characteristics of the FinnGen project eTable 2. Clinical characterization of patients with biallelic XXYLT1 variants eTable 3. Significant association of Recessive GWAS Intragenic lead SNPs with IRD eTable 4. Lead SNPs using an additive GWAS model eTable 5. Clinical characteristics of individuals homozygous for the XXYLT1 c.505-1G>C variant eTable 6. Summary of differentially expressed genes and their functions eReferences [file jamaophthalmol-e262795-s001.pdf]

Supplemental Online Content

Kraatari-Tiri M, Ishtiaq H, Tyrmi J, et al; FinnGen Project members. *XXYLT1* and mendelian retinal dystrophy. *JAMA Ophthalmol*. Published online July 30, 2026.
doi:10.1001/jamaophthalmol.2026.2795

eMethods

eResults

eDiscussion

eAppendix 1. Ethics declaration

eAppendix 2. Acknowledgments

eFigure 1. Flowchart of the study design

eFigure 2. Manhattan plot of the additive case-control GWAS

eFigure 3. Identification of rare XXYLT1 splice acceptor variant associated with IRD

eFigure 4. Ophthalmological findings

eFigure 5. Heatmap comparing gene expression profiles of XXYLT1 c.505-1G>C homozygous IRD patients and healthy controls, highlighting the top 30 differentially expressed genes (DEGs)

eTable 1. Case and control characteristics of the FinnGen project

eTable 2. Clinical characterization of patients with biallelic XXYLT1 variants

eTable 3. Significant association of Recessive GWAS Intragenic lead SNPs with IRD

eTable 4. Lead SNPs using an additive GWAS model

eTable 5. Clinical characteristics of individuals homozygous for the XXYLT1 c.505-1G>C variant

eTable 6. Summary of differentially expressed genes and their functions

eReferences

This supplemental material has been provided by the authors to give readers additional information about their work.

© 2026 American Medical Association. All rights reserved, including those for text and data mining, AI training, and similar technologies.

Supplementary methods

FinnGen Project

The FinnGen dataset comprises 520,210 individuals and aims to uncover associations between genetic variation and disease. Patient data were sourced from the Hospital Discharge Registry and the Cause of Death Registry.

In the FinnGen the endpoint ICD-10: H35.5 includes the ICD-10 diagnosis codes H35.50 through H35.54.

Fine mapping

Fine mapping was performed for all genomic regions in FinnGen where lead variants exceeded the genome-wide significance threshold ($p < 5 \times 10^{-8}$). All loci showing suggestive IRD associations were analyzed using a 4 Mb window (±2 Mb) centered on each index variant. To investigate the genomic context surrounding each locus, the Ensembl Genome Browser (https://www.ensembl.org/index.html) and DECIPHER were used. Each allele listed second for the lead SNPs (rs variants) in both models represents the alternative allele, which in this context corresponds to the disease-associated allele (Table 2).

Annotation of loci with biological functions and rare disease association

Genomic loci reaching genome-wide significance ($p < 5 \times 10^{-8}$) were manually annotated by evaluating candidate genes within each region. Biological functions were assessed using UniProt, and protein expression profiles were examined via The Human Protein Atlas. To identify potential associations with ophthalmological diseases, OMIM (https://www.omim.org/), ClinVar (https://www.ncbi.nlm.nih.gov/clinvar/?gr=1), GWAS Catalog (https://www.ebi.ac.uk/gwas/), and relevant literature were reviewed.

Oulu University Hospital IRD Cohort

Prior genetic testing conducted as part of diagnostic evaluation included single-gene or variant search (n = 9, 18%), gene panel (n = 24, 49%) and exome sequencing (n = 8, 16%). For 19 patients, no genetic testing had been performed.

All participants received comprehensive ophthalmological evaluations. Available medical records were reviewed, including clinical notes from slit-lamp examinations, best-corrected visual acuity measurements (Tomey TCP-3000P Polarising Chart Panel was used for patient 1, 3 and 4; for patients 2 and 5 the method is unknown), perimetry results, full-field electroretinography (ERG), and multimodal fundus imaging—comprising colour or pseudocolour images, fundus autofluorescence (FAF), optical coherence tomography (OCT), and fluorescein angiography.

UK IRD Cohort

Both participants underwent comprehensive ophthalmological examinations. Medical documentation was reviewed, including clinical records related to best-corrected visual acuity assessments (Snellen and LogMAR) as well as full-field ERG results and multimodal fundus imaging, comprising ultra-widefield pseudocolour and

© 2026 American Medical Association. All rights reserved, including those for text and data mining, AI training, and similar technologies.

autofluorescence imaging (Optos plc, Dunfermline, UK), and spectral-domain optical coherence tomography (SD-OCT; Heidelberg Spectralis, Heidelberg, Germany).

Sanger and whole genome sequencing

Sanger sequencing was performed to identify the *XXYLT1* c.505-1G>C variant in all 49 index patients using the ABI3500xL Genetic Analyzer system (Applied Biosystems) at Biocenter Oulu Sequencing Center. PCR amplification was carried out using forward primer (F) 'CCTTAGTGCGGCCTCAATTC' and reverse primer (R) 'GCAAAAGTCAGACACCCAGG'. Segregation analyses were similarly performed in participating family members of index cases homozygous for the *XXYLT1* c.505-1G>C variant.

Genomic DNA from 42 patients, including patient 3 (Figure 1; Family 3-II-1), was extracted using standard procedures for WGS (CeGaT, Tubingen, Germany).

A total of 100 ng of genomic DNA was used to construct sequencing libraries with the TruSeq DNA Nano Library Preparation Kit (Illumina, San Diego, CA), following the manufacturer's instructions. Sequencing was carried out on the NovaSeq X Plus platform (Illumina) using paired-end 2 × 151 bp reads. The resulting data yielded a Q30 score of ≥93.49%, indicating high base-calling accuracy. Sequencing reads were demultiplexed using bcl2fastq (v2.20, Illumina), and adapter sequences were trimmed using Skewer (v0.2.2).[1] No additional quality trimming was applied. All downstream analyses were performed using the Illumina DRAGEN Bio-IT Platform (v4.2.4).

High-quality sequencing reads were aligned to the human reference genome (GRCh38), and PCR duplicates were marked. Variant calling was performed using the default parameters of the Illumina DRAGEN's Bio-IT Platform, enabling the detection of small variants (SNVs and indels), structural variants, and regions of homozygosity. Copy number variants (CNVs) were identified using DRAGEN's self-normalization mode.

Raw sequencing read quality was assessed using FastQC, integrated within the DRAGEN platform. Comprehensive quality control summaries were compiled using MultiQC (v1.22.2, https://multiqc.info). Custom visualizations and statistical plots were generated in R (v4.0.4) utilizing the ggplot2 package.[2]

RNA extraction, NGS library preparation and sequencing

The total RNA was extracted using the RNeasy plus Mini Kit (Qiagen) from four patients homozygous for the *XXYLT1* c.505-1G>C variant and two healthy control individuals. For each sample, 1000ng of RNA was used to prepare libraries with the TruSeq Stranded mRNA Library Preparation Kit (Illumina), following the manufacturer's protocol. Library quantification and quality assessment were performed using the Bioanalyzer 2100 with High Sensitivity DNA Kit (Agilent), the Qubit Broad Range DNA-kit (Life Technologies), and qPCR-based NEBNext Library Quant Kit (NEB). All RNA samples exhibited high RNA integrity (RIN) values as follows: 10, 10, 10, 9.8, 7.8 and 9.8. Sequencing was conducted on the Illumina NextSeq550 platform in high-output mode using pair-ended 2 × 76 cycle reads, followed by FASTQ generation (Biocenter Oulu Sequencing Center).

© 2026 American Medical Association. All rights reserved, including those for text and data mining, AI training, and similar technologies.

RNA sequencing data analysis

The resulting mRNA-Seq data was analyzed using the DRAGEN RNA pipeline (v.4.4.4001) for gene expression quantification, with GRCh38 (hg38) as the reference genome. Differential gene expression analysis was subsequently performed using the DRAGEN Differential Expression module (v.4.3.7), comparing four homozygous index cases with two wild-type controls. A DESeq dataset was generated to identify differentially expressed genes. Given the markedly low expression of *XXYLT1* in homozygous carriers, the Integrative Genomics viewer (IGV) v.2.16.1 was used to manually inspect the aligned reads to assess potential aberrant splicing of *XXYLT1* transcripts relative to controls.

cDNA sequencing

Total RNA from skin fibroblast samples of three homozygous index cases and one wild-type control was reverse transcribed using the iScript cDNA synthesis Kit (Bio-Rad). Based on SpliceAI, the variant is predicted to cause loss of the canonical intron 1 acceptor site, which may lead to exon 2 skipping or an in-frame deletion of nine amino acids. To investigate these splicing outcomes, PCR amplification was performed using primers targeting *XXYLT1* exon 1 and exon 3, respectively:

Forward primer (exon 1): CCAAGTTCGAGGCGCACG
Reverse primer (exon 3): GGTCTAGGTCCAGCTGAATGA

© 2026 American Medical Association. All rights reserved, including those for text and data mining, AI training, and similar technologies.

Supplementary results

Clinical description of patients

Patient 1 (Table 1, Figure 1, eTable 2, eFigure 4): A 46-year-old Finnish male with homozygous *XXYLT1* c.505-1G>C variant presented with progressive central vision loss beginning in his mid-30s. Early findings included grainy macular pigmentation, later progressing to macular atrophy and large areas of hypoautofluorescence. Electrophysiology revealed both rod and cone dysfunction but normal VEP. Visual fields showed progressive central constriction and bilateral ring scotoma. Current visual acuity is 1.30 logMAR (20/400) in the right eye and 1.20 logMAR (20/320) in the left eye. The patient has mild posterior subcapsular cataractous changes and experiences night blindness. He meets the criteria of severe visual impairment and uses low-vision aids including a white cane.

Patient 2 (Table 1, Figure 1, eTable 2, eFigure 4): A 75-year-old Finnish male with a homozygous XXYLT1 c.505-1G>C variant was first evaluated at age 50 after reporting visual deterioration. Examination demonstrated initially cystoid macular edema. During follow-up, his visual acuities deteriorated, and peripheral pigmentary changes and macular puckering were observed on fundus examination. ERG showed reduced rod and cone amplitudes, and VEP revealed mildly delayed latencies. Bilateral cataract surgery was performed for nuclear cataracts. His current visual acuity is 0.20 logMAR (200/320) in the right eye and 0.20 logMAR (200/320) in the left eye, with bilateral ring scotoma. He has mild visual impairment and uses spectacles and low-vision aids.

Patient 3 (Table 1, Figure 1, Figure 3, eTable 2, eFigure 3): A 31-year-old Finnish female with homozygous *XXYLT1* c.505-1G>C variant was first examined at age 6 for decreased visual acuity. In the fundus there is grainy macular pigmentation with schisis-like changes and cysts, and autofluorescence demonstrates a hyperautofluorescent ring. ERG and VEP results were interpreted normal at age 16. Current visual acuity is 0.10 logMAR (16/20) in the right eye and 0.10 logMAR (16/20) in the left eye with preserved visual fields. She reports glare and progressive night vision difficulties. She does not meet the criteria of visual impairment and uses spectacles only.

Patient 4 (Table 1, Figure 1, eTable 2, eFigure 4): A 26-year-old Finnish female with homozygous *XXYLT1* c.505-1G>C variant presented with visual problems at 8 years of age and cystoid macular edema was detected first then. She is the sister of patient 3. Since 11 years, she has had bilateral chronic intermediary uveitis and secondary glaucoma which together with macular edema led to visual impairment at age 13 years. The patient underwent bilateral cataract surgery for secondary cataracts at age 14. The visual problems also include impaired contrast sensitivity and difficulties to see in the dark. ERG has demonstrated reduced responses. There is chronic cystoid macular edema, schisis-like changes, grainy pigmentation in the mid-periphery, and in the right eye there are glaucomatous defects. There is also posterior capsule opacification. Current visual acuity is 1.20 logMAR (20/320) in the right eye and 0.90 logMAR (20/160) in the left eye, with narrowed central fields. She has moderate visual impairment and uses low vision aids.

© 2026 American Medical Association. All rights reserved, including those for text and data mining, AI training, and similar technologies.

Patient 5 (Table 1, Figure 1, eTable 2, eFigure 4): A 51-year-old Finnish male with homozygous *XXYLT1* c.505-1G>C variant first noted visual decline in adulthood. Fundus imaging revealed papillomacular atrophy and central hypoautofluorescence. There was mild cystoid macular edema in the left eye. ERG revealed rod and cone dysfunction and delayed VEP latencies. Current visual acuity is 0.40 logMAR (20/50) in the right eye and 0.20 logMAR (20/32) in the left eye. In the temporal part of the central visual field of both right and left eye, there are defects that prohibit driving. He reports night blindness and headaches and has mild visual impairment managed with spectacles and magnifying devices.

Patient 6 (Table 1, Figure 1, eTable 2, eFigure 4): A 30-year-old male of Asian origin, homozygous for the *XXYLT1* c.766G>A, p.(Glu256Lys) variant reported poor central vision since childhood with initially preserved peripheral and night vision. Earlier evaluations showed bilateral foveal schisis progressing to macular outer retinal disruption with pigmentary mottling and hypoautofluorescence. Full-field ERG demonstrated marked macular dysfunction with mild generalized retinal involvement. Current visual acuity is 1.00 logMAR (20/200) in the right eye and 0.80 logMAR (20/125) in the left eye. He has severe visual impairment and uses low-vision aids.

Patient 7 (Table 1, Figure 1, eTable 2, eFigure 4): A 30-year-old male of Asian origin, a cousin of Patient 6, and homozygous for the same *XXYLT1* c.766G>A, p.(Glu256Lys) variant as Patient 6. He reported gradually progressive central visual decline. Imaging showed macular outer retinal disruption without schisis and central pigmentary mottling and hypoautofluorescence; the peripheral retina appeared unaffected. Full-field ERG indicated mild generalized dysfunction with severe macular involvement. Current visual acuity is 0.80 logMAR (20/125) in the right eye and 1.00 logMAR (20/200) in the left eye. He meets criteria for severe visual impairment but uses no visual aids and reports no additional ocular or systemic conditions.

GWAS with recessive model

Using a recessive model, GWAS identified 13 loci significantly associated with IRD reaching genome-wide significance ($p < 5 \times 10^{-8}$) (Figure 2). Among the lead SNPs from the recessive analysis, six intragenic lead SNPs were located within exonic regions or canonical splice sites (eTable 3). Notably, five of these variants corresponded to known pathogenic IRD-associated variants (Table 2).

Description of other previously unknown rare recessive loci

On chromosome 13, the lead variant rs1020045634 (GRCh38 13-110914511-G>A) is a rare variant (allele frequency, AF = 0.00486381), within a non-coding exon of a transcript of the *ANKRD10* gene. The imputation quality score for this variant is 0.89. In the FinnGen cohort, 793 individuals were heterozygous. Among homozygotes, fewer than five had a diagnosis corresponding to ICD-10 code H35.5. In the Finnish population reported in gnomAD v4.1.0, the AF for this variant is 0.0003735. The locus does not encompass any known IRD-associated genes. However, nearby lies Rho guanine nucleotide exchange factor (*ARHGEF7)* which has been proposed as a candidate gene based on evidence from various functional studies.

© 2026 American Medical Association. All rights reserved, including those for text and data mining, AI training, and similar technologies.

On chromosome 18, the lead variant rs529344081 (GRCh38 18-49086805-T>C) is located within an intron of the Dymeclin (*DYM)* gene. The imputation quality score for this variant is 0.84, with AF of 0.00205455 in FinnGen. In gnomAD v4.1.0, the AF is 0.003048 in the Finnish population and 0.00002945 in non-Finnish Europeans, while it is absent in other global populations. Fewer than five homozygous individuals for this variant were identified in FinnGen. *DYM* is associated with OMIM phenotypes Dyggve-Melchior-Clausen disease and Smith-McCort dysplasia. Nearby, *SMAD7* (Sma- and Mad-related protein 7), a protein-coding gene, has been proposed as a candidate gene based on evidence from functional studies.

On chromosome 20, the lead variant rs186466996 (GRCh38: 20-55873222 T>C) is a rare intergenic variant located near the *Cerebellin 4 Precursor* (*CBLN4*) gene. It has an imputation quality score of 0.97 and a low AF of 0.000588369 in the FinnGen cohort. This variant is not reported in gnomAD. Fewer than five homozygous individuals were identified in FinnGen, all of whom had a diagnosis corresponding to ICD-10 code H35.5. The locus does not encompass any known IRD-associated genes. However, nearby lies *Aurora kinase A* (*AURKA*) which has been proposed as a candidate gene based on its involvement in relevant molecular mechanisms.

Analysis of linkage disequilibrium between intergenic FinnGen lead variants and known pathogenic IRD founder variants in the genome sequenced IRD cohort

Seven of the lead SNPs identified in the recessive GWAS analysis were intronic or intergenic variants. Using WGS data from 42 IRD patients recruited at Oulu University Hospital, we confirmed linkage disequilibrium between rs1247877008 located near *GRM4* and the Finnish founder variant *TULP1* (NM_003322.6) c.148del,[3] observed in five alleles. Notably, one heterozygous carrier of the *TULP1* (NM_003322.6) c.148del variant did not possess the rs1247877008 variant, indicating a possible recombination event. Additionally, an association was confirmed between rs771075130 and the Finnish-enriched variant *CEP250* NM_007186.6 c.5959C>T, p.Gln1987*, identified in three alleles.

GWAS with additive model

GWAS was also performed using an additive model, identifying seven loci that reached genome-wide significance ($p < 5 \times 10^{-8}$) (eFigure 1, eTable 4). Of these, six loci included genes previously associated with IRD.

The lead variant rs150413925, located on chromosome 3 near the *RP11-171G7.2*, is an intergenic with an imputation quality score of 0.858. Its AF in FinnGen is 0.000297. According to gnomAD, the allele frequency is 0.00009420 in Finnish Europeans and 0.002639 in non-Finnish populations. Cell Adhesion Molecule 2 *(CADM2)* is the nearest protein-coding gene to this variant. Although *CADM2* is not currently associated with any OMIM-defined phenotypes, the GWAS catalogue reports its association with open angle glaucoma, refractive error, and pathological myopia. According to the Human Protein Atlas, CADM2 shows notably high expression in the retina (average nTPM: 92.0). In the FinnGen dataset, only 9 out of 307 individuals carrying the variant had a diagnosis corresponding to ICD-10 code H35.5 (hereditary retinal dystrophy), while the remaining

© 2026 American Medical Association. All rights reserved, including those for text and data mining, AI training, and similar technologies.

individuals were diagnosed with other eye disorders such as glaucoma, senile cataract, macular degeneration, and dry eye syndrome. These findings suggest that the signal observed in the Manhattan plot may represent a false-positive association with IRD. However, the potential role of cell adhesion molecules (CAMs) in retinal development should not be overlooked. CAMs are known to contribute to key neurodevelopmental processes, including cell fate determination, neuroblast migration, axon outgrowth, and synapse formation—mechanisms that may be relevant to retinal pathology.

© 2026 American Medical Association. All rights reserved, including those for text and data mining, AI training, and similar technologies.

Supplementary discussion

Within the chromosome 13 locus near the *ANKRD10* gene, *ARHGEF7* has emerged as a candidate gene is the Rho guanine nucleotide exchange factor. The GTEx database shows high expression levels of this gene in the retina (avg nTPM: 27.6). Although *ARHGEF7* is not currently linked to IRD, its association with retinal layer thickness has been reported in GWAS.[4] *ARHGEF7* encodes a guanine nucleotide exchange factor that activates RHOA, a small GTPase protein which plays a crucial role in maintaining the integrity and permeability of tight junctions and adherens junctions by regulating actin cytoskeleton dynamics. The integrity of these junctions is essential for the proper functioning of the retinal pigment epithelium and photoreceptor cells. The retina is particularly vulnerable to distruptions in pathways involving guanine nucleotide exchange factors (GEF) and RHOA activation. This sensitivity is underscored by retinal abnormalities observed in animal models with mutations in the related gene *ARHGEF18.*[5]

The candidate gene in the chromosome 18 locus near the *DYM* gene is Sma- and Mad-related protein 7 (*SMAD7*), which is involved in lens differentiation, periocular mesenchyme development, and retinal spatial patterning. Loss of *SMAD7* alters cell apoptosis and proliferation, influences BMP, TGF-β, and SHH signaling pathways, and leads to temporal changes in retinal neurogenesis highlighting its significant role in eye development.[6] Additionally, SMAD7 is targeted by miR-21, which modulates the expression of vascular endothelial growth factor (VEGF) under hyperglycemic conditions. This mechanism is particularly relevant to the pathogenesis of diabetic retinopathy.[7]

The candidate gene in the chromosome 20 locus near the *CBLN4* gene is Aurora A *(AURKA),* a key regulator of the cell cycle that plays a prominent role in cancer pathogenesis, particularly in RB1-deficient and MYCN-dysregulated tumors. An immunohistochemistry study of 67 patient specimens reported that AURKA is overexpressed in retinoblastoma (RB).[8] *AURKA* has also been implicated in pathways involving cilia, which are essential for photoreceptor function. Retinal photoreceptor degeneration is a common feature of various ciliopathies. Mutations in genes related to ciliary function, such as *RPGR* and *CEP290* contribute to both photoreceptor degeneration and syndromic ciliopathies.[9] Interactions between the prometastatic scaffolding protein HEF1/Cas-L/NEDD9 and Aurora A kinase at the basal body of cilia lead to phosphorylation and activation of HDAC6, a tubulin deacetylase that promotes ciliary disassembly.[10] AURKA's role in this process may indicate a link to retinal health, as impaired ciliary function can result in photoreceptor degeneration.

Ethics declaration

All studies were done in accordance with the Declaration of Helsinki. All participants provided informed consent for biobank research in accordance with the Finnish Biobank Act. Biobank protocols have been approved by Fimea and are used for participant recruitment. THL (approval number THL/2031/6.02.00/2017, amendments THL/1101/5.05.00/2017, THL/341/6.02.00/2018, THL/2222/6.02.00/2018, THL/1721/5.05.00/2019), and THL/283/6.02.00/2019, the Digital and Population Data Service Agency (VRK/4415/2019-3, VRK/6909/2018-3, and VRK43431/2017-3), the Social Insurance Institution (KELA) (KELA 58/522/2017, KELA 70/522/2019 and KELA 98/522/2019 and KELA 131/522/2018) and Statistics Finland (TK-53-1041-17) approved FinnGen study. The FinnGen study protocol Nr HUS/990/2017 is approved by the Coordinating Ethics Committee of the Hospital District of Helsinki and Uusimaa (HUS). Oulu University Hospital IRD Cohort study was approved by the Northern Ostrobothnia Hospital District (EETTMK: 45/2015, amendment 2024). The English cohort study was approved by the ethical review committee of Moorfields Eye Hospital and the Northwest London Research Ethics Committee (12/LO/0141), London, UK. All the participants signed written informed consent prior to participation in the research project.

Acknowledgments

We thank all study patients for participating in this study and European Retinal Disease Consortium (ERDC) network.

We acknowledge the participants and investigators of the FinnGen study. The FinnGen project is funded by two grants from Business Finland (HUS 4685/31/2016 and UH 4386/31/2016) and the following industry partners: AbbVie Inc., AstraZeneca UK Ltd, Biogen MA Inc., Bristol Myers Squibb Inc. (and Celgene Corporation & Celgene International II Sàrl), Genentech Inc., Merck Sharp & Dohme LCC, Pfizer Inc., GlaxoSmithKline Intellectual Property Development Ltd., Sanofi US Services Inc., Maze Therapeutics Inc., Johnson & Johnson Innovative Medicine Inc., Novartis AG, Boehringer Ingelheim International GmbH and Bayer AG. Following biobanks are acknowledged for delivering biobank samples to FinnGen: Auria Biobank (www.auria.fi/biopankki), THL Biobank (www.thl.fi/biobank), Helsinki Biobank (www.helsinginbiopankki.fi), Biobank Borealis of Northern Finland (https://www.ppshp.fi/Tutkimus-ja-opetus/Biopankki/Pages/Biobank-Borealis-briefly-in-English.aspx), Finnish Clinical Biobank Tampere (www.tays.fi/en-US/Research_and_development/Finnish_Clinical_Biobank_Tampere), Biobank of Eastern Finland (www.ita-suomenbiopankki.fi/en), Central Finland Biobank (www.ksshp.fi/fi-FI/Potilaalle/Biopankki), Finnish Red Cross Blood Service Biobank (www.veripalvelu.fi/verenluovutus/biopankkitoiminta), Terveystalo Biobank (www.terveystalo.com/fi/Yritystietoa/Terveystalo-Biopankki/Biopankki/) and Arctic Biobank (https://www.oulu.fi/en/university/faculties-and-units/faculty-medicine/northern-finland-birth-cohorts-and-arctic-biobank). All Finnish Biobanks are members of BBMRI.fi infrastructure (https://www.bbmri-eric.eu/national-nodes/finland/). Finnish Biobank Cooperative -FINBB (https://finbb.fi/) is the coordinator of BBMRI-ERIC operations in Finland. The Finnish biobank data can be accessed through the Fingenious® services

(https://site.fingenious.fi/en/) managed by FINBB. Biocenter Oulu Sequencing Center is acknowledged for providing RNA-Seq services and Sanger sequencing services.

This research was made possible through access to data in the National Genomic Research Library, which is managed by Genomics England Limited (a wholly owned company of the Department of Health and Social Care). The National Genomic Research Library holds data provided by patients and collected by the NHS as part of their care and data collected as part of their participation in research. The National Genomic Research Library is funded by the National Institute for Health Research and NHS England. The Wellcome Trust, Cancer Research UK. and the Medical Research Council have also funded research infrastructure.

eFigures

eFigure 1. Flowchart of the study design.

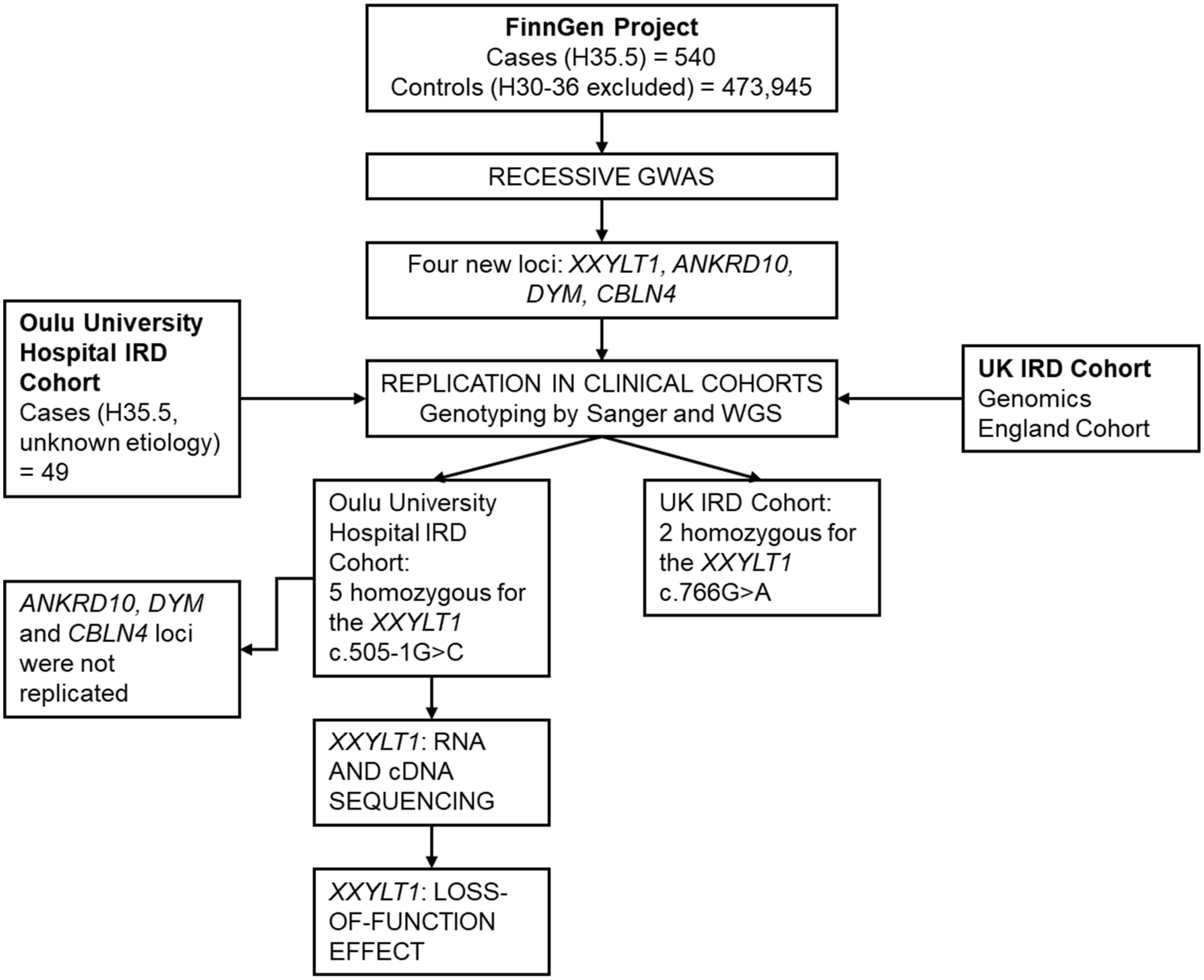

The figure outlines the analytical pipeline beginning with the FinnGen Project cohort, followed by a recessive genome-wide association study that identified four new loci. Subsequent replication was performed in clinical IRD cohorts using Sanger sequencing and whole-genome sequencing. In the Oulu University Hospital IRD cohort, five individuals were homozygous for *XXYLT1* c.505-1G>C, whereas the *ANKRD10*, *DYM*, and *CBLN4* loci did not replicate. In the UK IRD cohort, two individuals were homozygous for *XXYLT1* c.766G>A. Downstream analyses included RNA and cDNA sequencing of *XXYLT1*, demonstrating exon 2 skipping and a loss-of-function effect.

Abbreviations: GWAS = genome-wide association study, IRD = inherited retinal dystrophy, WGS = whole genome sequencing

eFigure 2. Manhattan plot of the additive case-control GWAS.

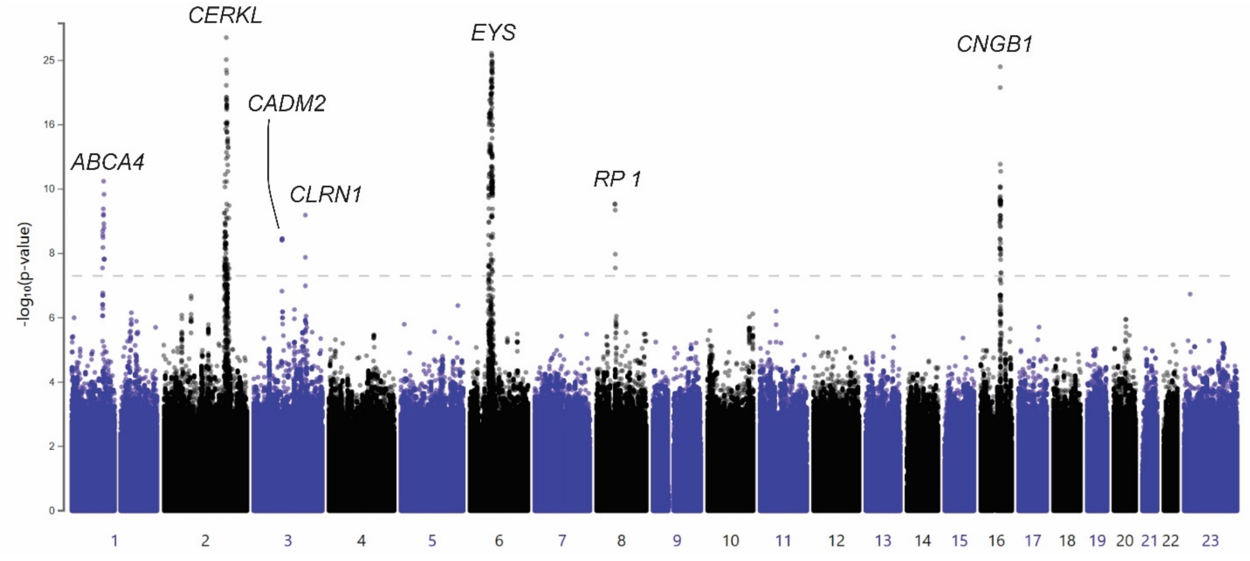

Chromosomal positions are displayed on the x-axis and –log10 (p-value) is plotted on the y-axis for each SNP. Seven loci reached genome-wide significance ($p < 5 \times 10^{-8}$). The candidate gene associated with each significant locus is labeled above the corresponding peak.

eFigure 3. Identification of rare XXYLT1 splice acceptor variant associated with IRD.

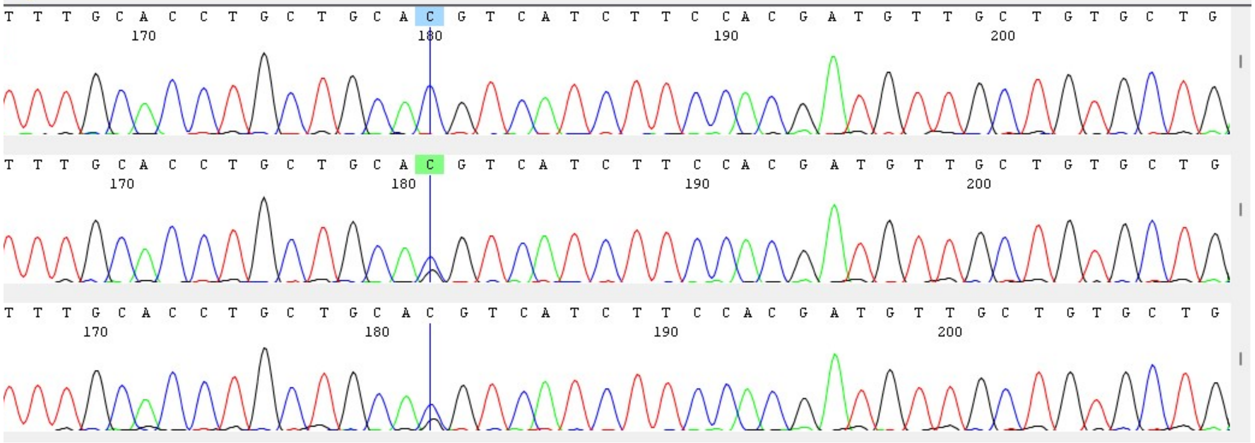

Sanger sequencing chromatograms showing the *XXYLT1* c.505-1G>C variant in a patient homozygous for the variant and diagnosed with IRD, alongside chromatograms from their heterozygous, unaffected parents.

eFigure 4. Ophthalmological findings.

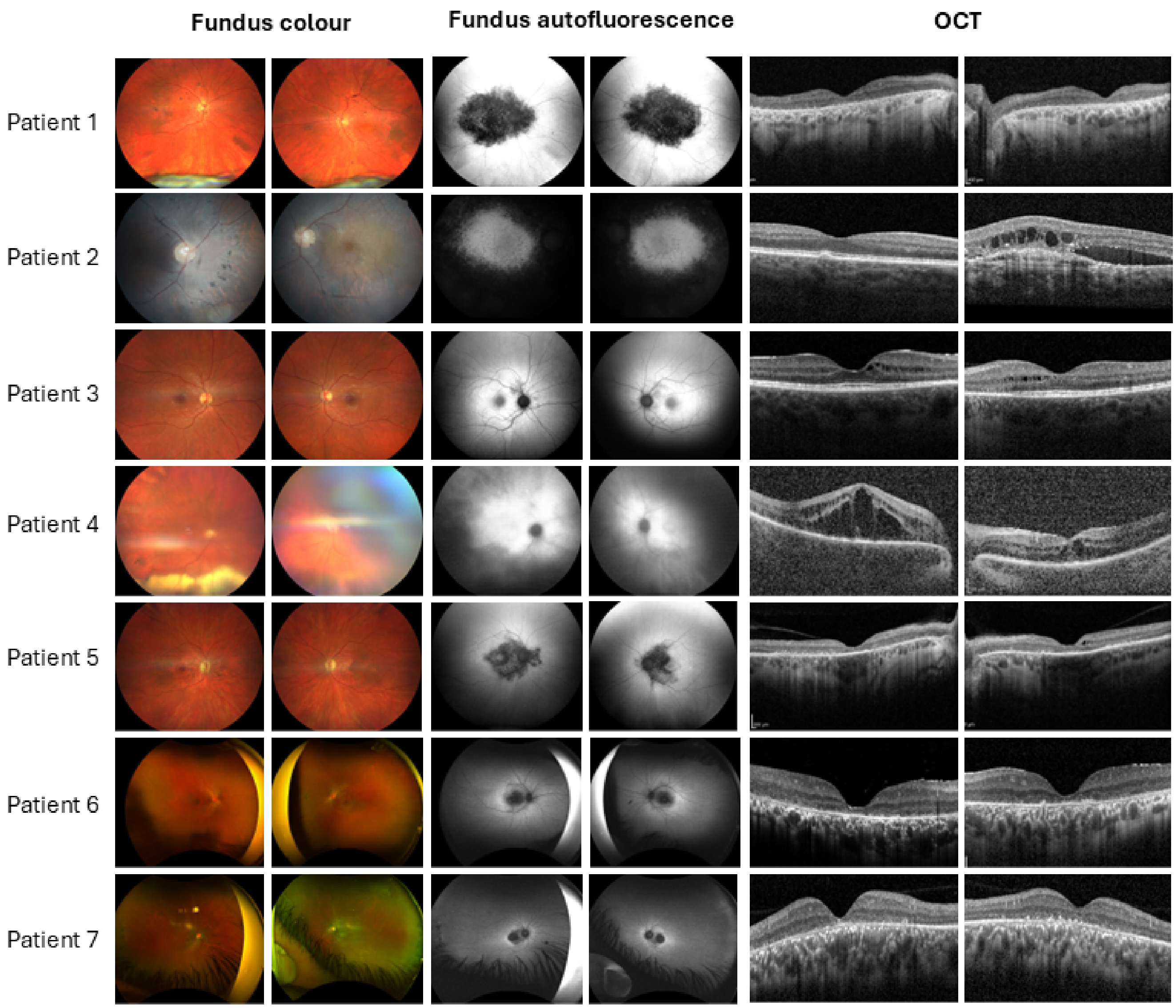

Multimodal retinal imaging for patients 1-7, including colour (Zeiss Clarus 700 or equivalent) or ultra-widefield pseudocolour (Optos plc, Dunfermline, UK) and fundus autofluorescence images, and macular optical coherence tomography (OCT) scans (Heidelberg Spectralis, Heidelberg Engineering, Heidelberg, Germany, and Topcon Corporation, Tokyo, Japan). Findings across most individuals are consistent with a macular, cone or cone-rod retinal dystrophy with fundus photographs showing macular lesions, cystoid macular oedema and/or schisis-like lesions in the fovea, retinal atrophy, pigment clumping in the mid-periphery, and pallor of the optic disc.

eFigure 5. Heatmap comparing gene expression profiles of *XXYLT1* c.505-1G>C homozygous IRD patients and healthy controls, highlighting the top 30 differentially expressed genes (DEGs).

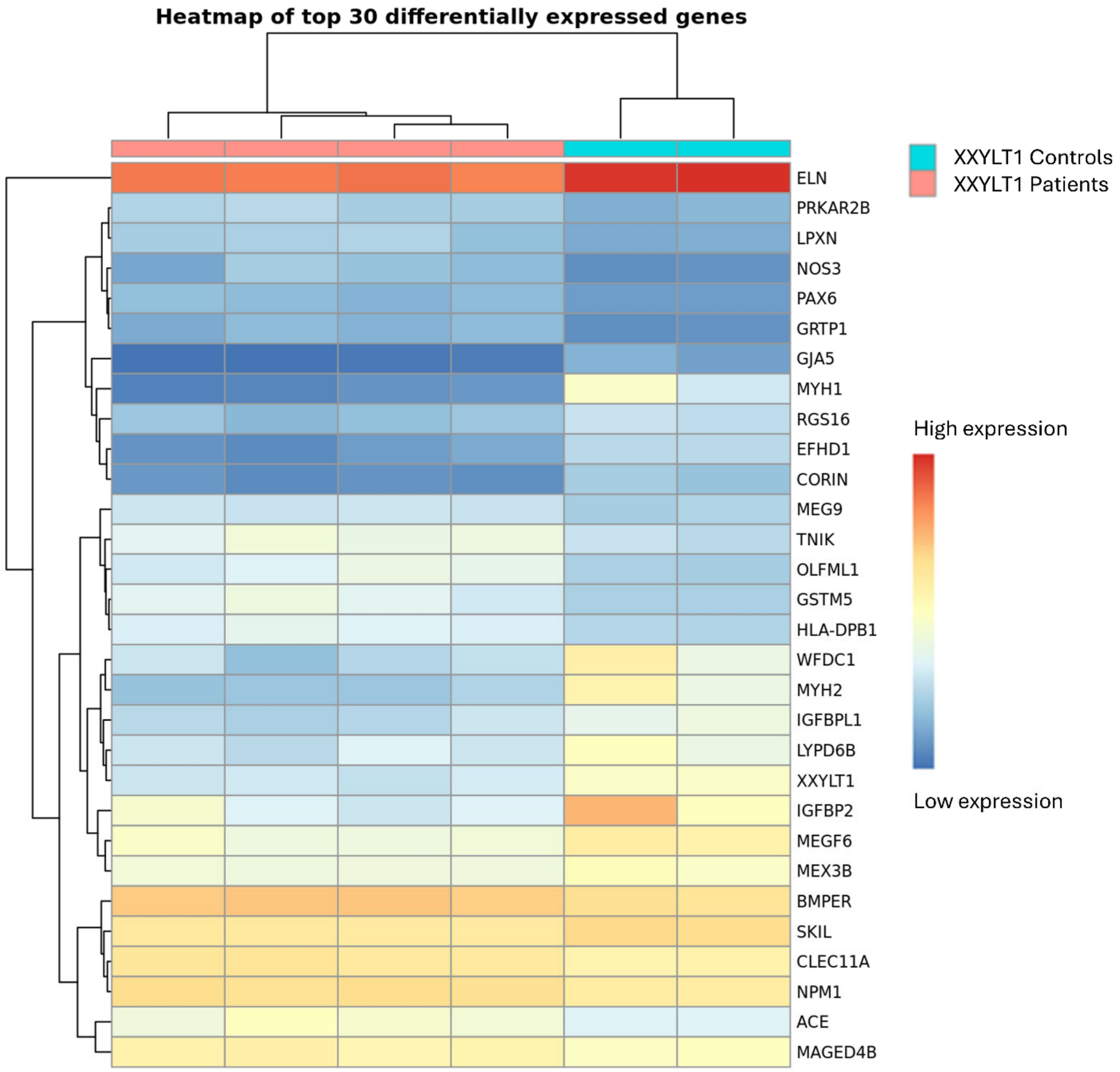

Among these DEGs, *XXYLT1* exhibited markedly reduced expression in homozygous IRD patients ($\log_2$ FC: -3.1, p-value: 9.0×10^{-23}). The heatmap also revealed differential expression of other genes, notably *TNIK* ($\log_2$ FC: 2.4, *p*-value: 1.9×10^{-4}) and *ELN* ($\log_2$ FC: –2.0, *p*-value: 1.2×10^{-15}), which may be indirectly involved in retinal dystrophy-related pathways. Colors on scale: top/red = high expression, blue/bottom low expression.

eTables

eTable 1. Case and control characteristics of the FinnGen project[a].

	**Cases (N=540)**	**Controls (N=473,945)**
Male, N (%)	251 (46 %)	203,931 (43 %)
Female, N (%)	289 (54 %)	270,014 (57 %)
Median age at first event (years) (IQR)	48.0 (33.8-80.3)	NA
Median age now (years) (IQR)	63.1 (49.2-75.4)	61.7 (45.8-73.7)

[a] The FinnGen case group comprised of 540 individuals.

eTable 2. Clinical characterization of patients with biallelic *XXYLT1* variants.

	**Patient 1 (F1-II-1)**	**Patient 2 (F2-II-1)**	**Patient 3 (F3-II-1)**	**Patient 4 (F3-II-3)**	**Patient 5 (F4-II-2)**	**Patient 6 (F5-III-1)**	**Patient 7 (F5-III-6)**
**Sex**	M	M	F	F	M	M	M
**Current age**	46 years	75 years	31 years	26 years	51 years	30 years	30 years
**Ancestry**	Finnish	Finnish	Finnish	Finnish	Finnish	Asian	Asian
**Variant detail (*XXYLT1* NM_152531.5)**	c.505-1G>C homozygous	c.505-1G>C homozygous	c.505-1G>C homozygous	c.505-1G>C homozygous	c.505-1G>C homozygous	c.766G>A; p.(Glu256Lys) homozygous	c.766G>A; p.(Glu256Lys) homozygous
**Age at first eye exam (years)**	35	50	6	8	>40	14	27
**First symptoms and findings**	Deterioration of vision, grainy pigmentation in the macula	Deterioration of vision, cystoid macular oedema	Compromised visual acuity, grainy pigmentation in the macula, normal night vision initially	Glasses from second grade, difficulties to see low contrasts, difficulties to see in the dark, deterioration of visual acuity, cystoid macular oedema	Deterioration of vision, atrophic degeneration in the maculae, mild macular oedema in his LE	Poor central vision since childhood, no problems with peripheral or night vision	Gradual deterioration in central vision
**Initial clinical diagnosis and ICD-10 codes**	H35.5 Cone-rod dystrophy	H35.5 Cone-rod dystrophy	H35.5 Stargardt disease	H35.5 Cone-rod dystrophy, H30.2 Panuveitis, H40.4 Glaucoma secondary to eye inflammation H35.9 unspecified retinal disorder, H26.2 complicated cataract, Z96.1 Pseudophakia	H35.5 hereditary macular dystrophy	H35.5, Retinal dystrophy	H35.5, Retinal dystrophy

eTable 2. Clinical characterization of patients with biallelic *XXYLT1* variants (continued).

	Patient 1 (F1-II-1) (continued)	Patient 2 (F2-II-1) (continued)	Patient 3 (F3-II-1) (continued)	Patient 4 (F3-II-3) (continued)	Patient 5 (F4-II-2) (continued)	Patient 6 (F5-III-1) (continued)	Patient 7 (F5-III-6) (continued)
**Clinical IRD diagnosis**	Cone-rod dystrophy	Cone-rod dystrophy	Cone-rod dystrophy	Cone-rod dystrophy	Cone-rod dystrophy	Cone-rod dystrophy	Cone-rod dystrophy
**BCVA (logMAR (Snellen))**	1.30 (20/400) / 1.20 (20/320)	0.20 (200/320) / 0.20 (200/320)	0.10 (16/20) / 0.10 (16/20),	1.20 (20/320) / 0.90 (20/160)	0.40 (20/50) / 0.20 (20/32)	1.00 (20/200) / 0.80 (20/125)	0.80 (20/125) / 1.00 (20/200)
**Fundus photography and OCT**	Grainy pigmentation in the macula, originally central hyperauto-flurescence, later turned to atrophy and a large area of hypoauto-fluorescence in both eyes, normal vasculature, discs slightly waxy, damaged neuroepithelium in the OCT	A few pigmentations and atrophy in the retinal periphery, retinal central hyperauto-fluorescence, cystoid macular oedema and macular puckering in both eyes, arterial narrowing and caliber changes	Schisis-like change in the macular area and single cysts in both eyes, normal optic disc, grainy looking macula, normal vasculature, hyperauto-fluorescence around fovea	Cystoid macular oedema RE and schisis-like change; LE alike but less marked macular findings; glaucomatous disc RE	Cystoid macular oedema LE, papillomacular atrophy in both eyes retinal central hypo-autofluorescence	First presented with bilateral foveal schisis, later progressed to outer retinal disruption involving the macula. Central macula pigmen-tary mottling and hypoautofluoresc ence, with altered autofluorescence at the posterior pole. The peripheral retina appears relatively uninvolved with no pigment spicules noted	Outer retinal disruption involving the macula, no macular schisis noted. Central macula pigmentary mottling and hypoauto-fluorescence, with altered autofluorescence at the posterior pole. The peripheral retina appears relatively uninvolved with no pigment spicules noted
**ERG and VEP**	ERG age 39: low amplitudes suggesting rod and cone dysfunction	ERG age 71: low amplitudes suggesting rod and cone dysfunction	ERG age 16: normal	ERG age 11: low amplitudes, especially scotopic	ERG age 50: both scotopic and photopic responses are reduced, the photopic cone response latencies also prolonged	ERG age 18: marked bilateral macular dysfunction, mild generalized rod and cone system dysfunction, normal bright flash b:a ratio	Severe wide-spread macular dysfunction bilaterally, full field ERGs suggest very mild loss of retinal function bilaterally

eTable 2. Clinical characterization of patients with biallelic *XXYLT1* variants (continued).

	Patient 1 (F1-II-1) (continued)	Patient 2 (F2-II-1) (continued)	Patient 3 (F3-II-1) (continued)	Patient 4 (F3-II-3) (continued)	Patient 5 (F4-II-2) (continued)	Patient 6 (F5-III-1) (continued)	Patient 7 (F5-III-6) (continued)
**VEP**	VEP age 39: normal	VEP age 71: slightly delayed latencies	VEP age 16: normal	VEP age 11: normal	VEP age 50: uniformly pro-longed latencies and reduced amplitudes on both sides	NA	NA
**Refractive error (before cataract surgery)**	RE: -0.50 cyl+0.50 ax 140<br>LE: -0.75 cyl +0.75 ax 10	RE:±0<br>LE:-0.75 cyl +0.75 ax 0	RE: +1.75<br>LE: +1.75	RE: -0.25 cyl +1.75 ax 90<br>LE: -0.25 cyl +1.75 ax 105	RE: -0.25<br>LE: -0.5 cyl -0.25 ax70	RE: +1.00 cyl-0.75 ax 90<br>LE: +1.75 cyl-1.75 ax 35	NA
**Visual field**	Central 5° long remaining (RE), bilateral ring scotoma of 40° (absolute sco-toma in the LE), advanced to large absolute scotoma of 40° in diameter similarly in the RE and the LE	Central 5 degrees of field remain, as well as a seeing area 30-60 degrees below and temporal of fixation in both RE and LE	Normal	Low sensitivity in the central field, narrowing of the nasal and superior fields, temporal fields intact	Absolute visual field defect in the temporal part of the central field in both eyes, normal peripheral fields, driving no longer allowed	NA	NA
**Cataract**	Tiny posterior subcapsular cataractous dots	Nuclear; cataract surgery in both eyes	No	Posterior capsule opacification; cataract surgery in both eyes	No	No	No

eTable 2. Clinical characterization of patients with biallelic *XXYLT1* variants (continued).

	Patient 1 (F1-II-1) (continued)	Patient 2 (F2-II-1) (continued)	Patient 3 (F3-II-1) (continued)	Patient 4 (F3-II-3) (continued)	Patient 5 (F4-II-2) (continued)	Patient 6 (F5-III-1) (continued)	Patient 7 (F5-III-6) (continued)
**Additional ocular symptoms**	Night blindness	Night blindness, glare	Glare, progressive night vision problems, symptoms of dry eye, exophoria	Intermediary uveitis, secondary glaucoma in both eyes, secondary cataract, deterioration of colour vision, night blindness, glare	Night blindness, headaches	No	No
**Classification of visual impairment**	Severe	Mild	No	Moderate	Mild	Severe	Severe
**Treatment, aids**	White cane, low vision aids	Spectacles, low vision aids	Spectacles	Low vision aids, uveitis treatment, glaucoma treatment	Spectacles, low vision aids (magnifying glasses)	Low vision aids	No
**Other health problems**	No	Mitral valve prolapse, hypertension, atrial fibrillation, hyperlipidemia, benign prostatic hyperplasia	No	No	Fallot tetralogy operated as a child	Sleep apnea	No

Abbreviations: ERG = electroretinogram, F = female, M = male, OCT = optical coherence tomography, VEP = visual evoked potential, RE = right eye, LE = left eye.

eTable 3. Significant association of Recessive GWAS Intragenic lead SNPs with IRD.

Lead variant rsID	EAF	Number of heterozygotes in cases (% from all cases) / controls (% from all controls)	Consequence of the lead variant	ACMG/AMP classification[a] and used criteria	PMID if the variant has previously been published
rs200711686	0.00555	20 (3.7%) / <5 (<0.001%)	NM_201548.5(CERKL):c.375C>G, p.(Cys125Trp)	Pathogenic (PS4_strong, PM2_supporting, PM3_moderate, PP1_strong)	20554613, 27208204, 29068140, 40571344
rs201922399	0.00507	7 (1.3%) / 5 (0.001%)	NM_152531.5(XXYLT1): c.505-1G>C	NA, disease gene	This study
rs528919874	0.00714	22 (4.1%) / 9 (0.002%)	NM_001142800.2(EYS):c.8648_8655del, p.(Thr2883Lysfs*4)	Likely pathogenic (PS4_strong, PM2_supporting, PP1_strong)	20537394, 28704921, 29159838, 40571344
rs397515360	0.00271	5 (0.9%) / <5 (<0.9%)	NM_019098.5(CNGB3):c.1148del p.(Thr383Ilefs*13)	Pathogenic (PVS1_very strong, PM2_supporting, PP1_strong)	28746191, 29769798, 15657609, 40571344
rs121918284	0.00202	<5 (<0.9%) / <5 (<0.001%)	NM_004183.4(BEST1):c.422G>A p.(Arg141His)	Pathogenic (PM2_supporting, PM3_moderate, PM5_moderate, PP1_strong, PP3_strong)	10854112, 32531858, 33302512, 33369172, 34015078,
rs201162411	0.00625	18 (3.3%) / 14 (0.003%)	NM_001297.5(CNGB1):c.2957A>T p.(Asn986Ile)	Pathogenic (PS4_strong, PM2_supporting, PP3_supporting, PP1_strong)	21147909, 28056120, 23105016, 26355662, 28559085, 29912909, 31725169, 40571344

[a] The American College of Medical Genetics and Association for Molecular Pathology (ACMG/AMP) variant interpretation guidelines were used to classify the variants[11].

Abbreviations: NA, not applicable.

eTable 4. Lead SNPs using an additive GWAS model (previously unknown additive locus in bold).

Lead variant (GRCh38)[a]	Lead variant rsID	EAF	P value	Number of heterozygotes in cases (% from all cases) / controls (% from all controls)[b]	Nearest gene[c]	Candi-date gene[d]	Type	Pheno-type MIM number	ClinVar variation ID	Current Status of the variant	Lit. ref.
1:92999764:C>T	rs924187278	0.000564	2.0×10^{-9}	11 (2.0%) / 558 (0.01%)	*MTF2*	*ABCA4*	Intergenic variant	153800, 604116, 248200, 248200, 601718, 248200	NA	Nearby known pathogenic	12
2:181603943:G>C	rs200711686	0.00554	2.5×10^{-30}	15 (2.8%) / 4992 (1.1%)	*CERKL*	*CERKL*	Missense	608380	866659	Known pathogenic	13
3:83682513:T>C	rs150413925	0.000337	3.5×10^{-9}	7 (1.3%) / 279 (0.06%)	*CADM2*	***CADM2***	Intergenic variant	NA	NA	This study[e]	NA
3:150928107:A>C	rs121908140	0.00453	6.4×10^{-10}	6 (1.1%) / 4215 (0.9%)	*CLRN1*	*CLRN1*	Nonsense	614180, 276902	4392	Known pathogenic	14
6:63721375: TTCTGCATG>T	rs528919874	0.00636	3.3×10^{-27}	11 (2.0%) / 6550 (1.4%)	*EYS*	*EYS*	Nonsense	602772	550019	Known pathogenic	15
8:54523639:C>T	rs1345244391	0.000587347	2.9×10^{-10}	8 (1.5%) / 427 (0.09%)	*RP1*	*RP1*	Intron	180100	NA	Nearby known pathogenic	16
16:57901371:T>A	rs201162411	0.00624219	5.5×10^{-24}	12 (2.2%) / 5701 (1.2%)	*CNGB1*	*CNGB1*	Missense	613767	166891	Known pathogenic	17

[a] Lead variant, chromosome, base-pair position, reference allele and effect allele (formatted as "chromosome:position:reference>effect").

[b] Number of homozygotes in cases (% of all cases) / controls (% of all controls), the count and proportion of homozygous individuals among IRD cases (N = 540) and controls (N = 473,945).

[c] Nearest gene is the closest protein coding gene to the lead variant.

[d] Candidate gene refers to the gene presumed to drive the association signal based on functional evidence and prior literature.

[e] This study identified no effect allele for CADM2 variant in independent cohorts.

Abbreviations: EAF, effect allele frequency; NA, not available.

eTable 5. Clinical characteristics of individuals homozygous for the *XXYLT1* c.505-1G>C variant.

Clinical characteristics	N (%)
Male	9 (47 %)
Female	10 (53 %)
Average age now	57 years
Diagnosis of hereditary retinal or macular dystrophy	10 (53 %)[a]
Average age at the diagnosis, years (SD)	39 (18.3)
Median age at the diagnosis, years	38
Cataract	7 (37 %)

[a] Most of those *XXYLT1* c.505-1G>C homozygous individuals who did have the diagnosis of hereditary retinal or macular dystrophy also had other eye-related diagnosis including diplopia, age-related macular degeneration, diabetic maculopathy/retinopathy, visual disturbances, visual impairment, and retinal detachment.

eTable 6. Summary of differentially expressed genes and their functions.

Gene	Gene name	log_2 FC	p-value adjusted	Gene function
**XXYLT1**	Xyloside Xylosyltransferase 1	-3.1	$9.0x10^{-23}$	XXYLT1 adds a second xylose sugar to specific proteins like Notch receptors, modifying them for proper cellular signaling. It is a causal candidate gene for Inherited Retinal Dystrophy (this study).
ELN	Elastin	-2.0	$1.1x10^{-15}$	ELN is responsible for elasticity and resilience to tissues like skin, lungs, and blood vessels. Mutation can affect connective tissues, including those in the eye, involving ocular hypertension or vascular anomalies.
MYH1	Myosin Heavy Chain 1	-7.8	$5.0x10^{-13}$	MYH1 is involved in skeletal muscle contraction, especially in fast-twitch (type IIx) muscle fibers.
MYH2	Myosin Heavy Chain 2	-5.1	$1.3x10^{-11}$	MYH2 participates in skeletal muscle contraction, especially in fast-twitch (type IIa) muscle fibers
GSTM5	Glutathione S-Transferase Mu 5	2.8	$1.1x10^{-5}$	GSTM5 detoxify and eliminate harmful compounds such as environmental toxins, drugs, and carcinogens with the help of glutathione. Variants in this gene may influence susceptibility to glaucoma and oxidative stress-related retinal diseases.
CORIN	Corin, Serine Peptidase	-3.9	$1.4x10^{-5}$	Corin participates in the activation of cardiac enzymes, i.e., atrial natriuretic peptide and brain natriuretic peptide to maintain blood pressure and fluid balance. It may influence ocular health.
HLA-DPB1	Major Histocompatibility Complex, Class II, DP Beta 1	2.2	$2.9x10^{-5}$	HLA-DPB1 helps to trigger the immune system in response to bacterial or viral peptide exposure. It is involved in autoimmune eye diseases like uveitis and sarcoidosis-related ocular inflammation.
MEGF6	Multiple EGF-Like Domains 6	-2.4	$4.2x10^{-5}$	MEGF6 participates in vesicle-mediated transport and calcium ion binding. Its expression in vascular tissues suggests a potential role in ocular vasculature, though its main biological function is still unclear.
CLEC11A	C-Type Lectin Domain Containing 11A	1.0	$4.5x10^{-5}$	CLEC11A participates in the development of hematopoietic stem cells and promotes bone formation

eTable 6. Summary of differentially expressed genes and their functions (continued).

Gene (continued)	Gene name (continued)	log_2 FC (continued)	p-value adjusted (continued)	Gene function (continued)
GJA5	Gap Junction Protein Alpha 5	-5.5	8.7×10^{-5}	GJA5 encodes connexin 40 that allows communication among heart cells and maintains normal cardiac rhythm forming gap junctions. Connexins are also important for lenses and retina.
IGFBPL1	Insulin-Like Growth Factor Binding Protein-Like 1	-2.5	8.8×10^{-5}	IGFBPL1 helps regulate insulin-like growth factor activity in survival, cell growth, and differentiation. It may influence retinal development and axon guidance suggesting its role in retinal ganglion cell survival.
OLFML1	Olfactomedin Like 1	2.8	1.0×10^{-4}	OLFML1 belongs to the olfactomedin family, and olfactomedin proteins are involved in eye development, especially in the trabecular meshwork. OLFML1's role is still under investigation.
TNIK	TRAF2 and NCK Interacting Kinase	2.4	1.8×10^{-4}	TNIK participates in Wnt signaling, a pathway for development, cell growth, and cancer progression.
GRTP1	Growth Hormone Regulated TBC Protein 1	3.6	1.9×10^{-4}	GRTP1 participates in regulating intracellular trafficking due to growth hormone signaling and may affect retinal function.
BMPER	BMP Binding Endothelial Regulator	1.0	2.4×10^{-4}	BMPER acts in regulating bone morphogenetic protein (BMP) signaling, which is crucial for bone and cartilage development. It may also be crucial for ocular morphogenesis.
PAX6	Paired Box 6	2.4	2.6×10^{-4}	PAX6 is a key regulator of brain and eye development. It controls the expression of other specific genes involved in forming the eyes, pancreas, and central nervous system during embryogenesis.
WFDC1	WAP Four-Disulfide Core Domain 1	-4.3	2.8×10^{-4}	WFDC1 may influence corneal structure and immune regulation in the eye and associated with corneal staphyloma and ocular hypotension

eTable 6. Summary of differentially expressed genes and their functions (continued).

Gene (continued)	Gene name (continued)	$\log_2$ FC (continued)	p-value adjusted (continued)	Gene function (continued)
NOS3	Nitric Oxide Synthase 3	4.0	2.9×10^{-4}	NOS3 encodes endothelial nitric oxide synthase that produces nitric oxide to enhance blood vessel relaxation and control vascular function. It regulates ocular blood flow and may be involved in retinopathy of prematurity (ROP) and glaucoma.
RGS16	Regulator of G Protein Signaling 16	-2.2	3.5×10^{-4}	RGS16 plays a role in signal transduction by regulating G protein signalling and it is also associated with age-related macular degeneration.
PRKAR2B	Protein Kinase cAMP-Dependent Type II Regulatory Subunit Beta	2.4	3.7×10^{-4}	PRKAR2B helps mediate cellular responses to cAMP, influencing energy balance, metabolism, and gene regulation, and may affect retinal cell signalling.

Supplementary references

1. Jiang H, Lei R, Ding SW, Zhu S. Skewer: a fast and accurate adapter trimmer for next-generation sequencing paired-end reads. *BMC Bioinformatics*. 2014;15:182. doi:10.1186/1471-2105-15-182

2. Wickham H. *Ggplot2: Elegant Graphics for Data Analysis*. Springer; 2009. doi:10.1007/978-0-387-98141-3

3. Hagstrom SA, North MA, Nishina PL, Berson EL, Dryja TP. Recessive mutations in the gene encoding the tubby-like protein TULP1 in patients with retinitis pigmentosa. *Nat Genet*. 1998;18(2):174-176. doi:10.1038/ng0298-174

4. Jackson VE, Wu Y, Bonelli R, et al. Multi-omic spatial effects on high-resolution AI-derived retinal thickness. *Nat Commun*. 2025;16(1):1317. doi:10.1038/s41467-024-55635-7

5. Arno G, Carss KJ, Hull S, et al. Biallelic Mutation of ARHGEF18, Involved in the Determination of Epithelial Apicobasal Polarity, Causes Adult-Onset Retinal Degeneration. *Am J Hum Genet*. 2017;100(2):334-342. doi:10.1016/j.ajhg.2016.12.014

6. Zhang R, Huang H, Cao P, Wang Z, Chen Y, Pan Y. Sma- and Mad-related protein 7 (Smad7) is required for embryonic eye development in the mouse. *J Biol Chem*. 2013;288(15):10275-10285. doi:10.1074/jbc.M112.416719

7. Haque R, Iuvone PM, He L, et al. The MicroRNA-21 signaling pathway is involved in prorenin receptor (PRR) -induced VEGF expression in ARPE-19 cells under a hyperglycemic condition. *Mol Vis*. 2017;23:251-262.

8. Borah NA, Mittal R, Sucharita S, et al. Aurora Kinase A Is Overexpressed in Human Retinoblastoma and Correlates with Histopathologic High-Risk Factors: Implications for Targeted Therapy. *Am J Pathol*. 2024;194(9):1780-1798. doi:10.1016/j.ajpath.2024.05.006

9. Rachel RA, Li T, Swaroop A. Photoreceptor sensory cilia and ciliopathies: focus on CEP290, RPGR and their interacting proteins. *Cilia*. 2012;1(1):22. doi:10.1186/2046-2530-1-22

10. Pugacheva EN, Jablonski SA, Hartman TR, Henske EP, Golemis EA. HEF1-dependent Aurora A activation induces disassembly of the primary cilium. *Cell*. 2007;129(7):1351-1363. doi:10.1016/j.cell.2007.04.035

11. Richards S, Aziz N, Bale S, et al. Standards and guidelines for the interpretation of sequence variants: a joint consensus recommendation of the American College of Medical Genetics and Genomics and the Association for Molecular Pathology. *Genet Med*. 2015;17(5):405-424. doi:10.1038/gim.2015.30

12. Rozet JM, Gerber S, Souied E, et al. Spectrum of ABCR gene mutations in autosomal recessive macular dystrophies. *Eur J Hum Genet*. 1998;6(3):291-295. doi:10.1038/sj.ejhg.5200221

13. Tuson M, Marfany G, Gonzàlez-Duarte R. Mutation of CERKL, a novel human ceramide kinase gene, causes autosomal recessive retinitis pigmentosa (RP26). *Am J Hum Genet*. 2004;74(1):128-138. doi:10.1086/381055

14. Khan MI, Kersten FFJ, Azam M, et al. CLRN1 mutations cause nonsyndromic retinitis pigmentosa. *Ophthalmology*. 2011;118(7):1444-1448. doi:10.1016/j.ophtha.2010.10.047

15. Abd El-Aziz MM, Barragan I, O'Driscoll CA, et al. EYS, encoding an ortholog of Drosophila spacemaker, is mutated in autosomal recessive retinitis pigmentosa. *Nat Genet*. 2008;40(11):1285-1287. doi:10.1038/ng.241

16. Sullivan LS, Heckenlively JR, Bowne SJ, et al. Mutations in a novel retina-specific gene cause autosomal dominant retinitis pigmentosa. *Nat Genet*. 1999;22(3):255-259. doi:10.1038/10314

17. Bareil C, Hamel CP, Delague V, Arnaud B, Demaille J, Claustres M. Segregation of a mutation in CNGB1 encoding the beta-subunit of the rod cGMP-gated channel in a family with autosomal recessive retinitis pigmentosa. *Hum Genet*. 2001;108(4):328-334. doi:10.1007/s004390100496
