# Supplementary material for: XXYLT1 and Mendelian Retinal Dystrophy
Source: JAMA Ophthalmol. 2026 Jul 30:e262795. Online ahead of print. doi: 10.1001/jamaophthalmol.2026.2795 (PMC13425242; doi:10.1001/jamaophthalmol.2026.2795)
Supplement: Supplement 2. — FinnGen Project members [file jamaophthalmol-e262795-s002.pdf]

Supplemental Online Content: Nonauthor Collaborators

*Indicates required information. Only first name, last name, and suffix will appear in PubMed.

| **Group Name:*** | | | | | | |
|---|---|---|---|---|---|---|
| First Name and Middle Initial(s)* | Last Name* | Suffix* | Academic Degrees | Institution and location (city, state/province, country) | Role or Contribution, eg, chair, principal investigator | Subgroup, eg, Steering Committee |
| Aarno | Palotie | | | Institute for Molecular Medicine Finland (FIMM), HiLIFE, University of Helsinki, Helsinki, Finland; Broad Institute of MIT and Harvard; Massachusetts General Hospital | Steering Committee | Steering Committee |
| Mark | Daly | | | Institute for Molecular Medicine Finland (FIMM), HiLIFE, University of Helsinki, Helsinki, Finland; Broad Institute of MIT and Harvard; Massachusetts General Hospital | Steering Committee | Steering Committee |
| Bridget | Riley-Gills | | | Abbvie, Chicago, IL, United States | Steering Committee | Pharmaceutical companies |
| Howard | Jacob | | | Abbvie, Chicago, IL, United States | Steering Committee | Pharmaceutical companies |
| Coralie | Viollet | | | Astra Zeneca, Cambridge, United Kingdom | Steering Committee | Pharmaceutical companies |
| Slavé | Petrovski | | | Astra Zeneca, Cambridge, United Kingdom | Steering Committee | Pharmaceutical companies |
| Alix | Berton | | | Bayer AG, Leverkusen, Germany | Steering Committee | Pharmaceutical companies |
| Santha | Ramakrishnan | | | Bayer AG, Leverkusen, Germany | Steering Committee | Pharmaceutical companies |

Supplemental Online Content: Nonauthor Collaborators

*Indicates required information. Only first name, last name, and suffix will appear in PubMed.

| First Name and Middle Initial(s)* | Last Name* | Suffix* | Academic Degrees | Institution and location (city, state/province, country) | Role or Contribution, eg, chair, principal investigator | Subgroup, eg, Steering Committee |
|---|---|---|---|---|---|---|
| Ellen | Tsai | | | Biogen, Cambridge, MA, United States | Steering Committee | Pharmaceutical companies |
| Zhihao | Ding | | | Boehringer Ingelheim, Ingelheim am Rhein, Germany | Steering Committee | Pharmaceutical companies |
| Emily | Holzinger | | | Bristol Myers Squibb, New York, NY, United States | Steering Committee | Pharmaceutical companies |
| Robert | Plenge | | | Bristol Myers Squibb, New York, NY, United States | Steering Committee | Pharmaceutical companies |
| Joseph | Maranville | | | Bristol Myers Squibb, New York, NY, United States | Steering Committee | Pharmaceutical companies |
| Mark | McCarthy | | | Genentech, San Francisco, CA, United States | Steering Committee | Pharmaceutical companies |
| Rion | Pendergrass | | | Genentech, San Francisco, CA, United States | Steering Committee | Pharmaceutical companies |
| Jonathan | Davitte | | | GlaxoSmithKline, Collegeville, PA, United States | Steering Committee | Pharmaceutical companies |
| Chia-Yen | Chen | | | Merck, Kenilworth, NJ, United States | Steering Committee | Pharmaceutical companies |
| Melis Atalar | Aksit | | | Pfizer, New York, NY, United States | Steering Committee | Pharmaceutical companies |
| Anna | Vlahiotis | | | Pfizer, New York, NY, United States | Steering Committee | Pharmaceutical companies |

Supplemental Online Content: Nonauthor Collaborators

*Indicates required information. Only first name, last name, and suffix will appear in PubMed.

| First Name and Middle Initial(s)* | Last Name* | Suffix* | Academic Degrees | Institution and location (city, state/province, country) | Role or Contribution, eg, chair, principal investigator | Subgroup, eg, Steering Committee |
|---|---|---|---|---|---|---|
| Katherine | Klinger | | | Translational Sciences, Sanofi R&D, Framingham, MA, USA | Steering Committee | Pharmaceutical companies |
| Clement | Chatelain | | | Translational Sciences, Sanofi R&D, Framingham, MA, USA | Steering Committee | Pharmaceutical companies |
| Jorg | Blankenstein | | | Translational Sciences, Sanofi R&D, Framingham, MA, USA | Steering Committee | Pharmaceutical companies |
| Karol | Estrada | | | Maze Therapeutics, San Francisco, CA, United States | Steering Committee | Pharmaceutical companies |
| Robert | Graham | | | Maze Therapeutics, San Francisco, CA, United States | Steering Committee | Pharmaceutical companies |
| Dawn | Waterworth | | | Johnson & Johnson Innovative Medicine, Spring House, PA, United States | Steering Committee | Pharmaceutical companies |
| Chris | O´Donnell | | | Novartis Institutes for BioMedical Research, Cambridge, MA, United States | Steering Committee | Pharmaceutical companies |
| Nicole | Renaud | | | Novartis Institutes for BioMedical Research, Cambridge, MA, United States | Steering Committee | Pharmaceutical companies |
| Tomi P. | Mäkelä | | | HiLIFE, University of Helsinki, Finland, Finland | Steering Committee | University of Helsinki & Biobanks |

Supplemental Online Content: Nonauthor Collaborators

*Indicates required information. Only first name, last name, and suffix will appear in PubMed.

| First Name and Middle Initial(s)* | Last Name* | Suffix* | Academic Degrees | Institution and location (city, state/province, country) | Role or Contribution, eg, chair, principal investigator | Subgroup, eg, Steering Committee |
|---|---|---|---|---|---|---|
| Jaakko | Kaprio | | | Institute for Molecular Medicine Finland (FIMM), HiLIFE, University of Helsinki, Helsinki, Finland | Steering Committee | University of Helsinki & Biobanks |
| Minna | Ruddock | | | Arctic biobank / University of Oulu | Steering Committee | University of Helsinki & Biobanks |
| Lila | Kallio | | | Auria Biobank / University of Turku / Wellbeing Services County of Southwest Finland, Turku, Finland | Steering Committee | University of Helsinki & Biobanks |
| Antti | Hakanen | | | Auria Biobank / University of Turku / Wellbeing Services County of Southwest Finland, Turku, Finland | Steering Committee | University of Helsinki & Biobanks |
| Terhi | Kilpi | | | THL Biobank / Finnish Institute for Health and Welfare (THL), Helsinki, Finland | Steering Committee | University of Helsinki & Biobanks |
| Markus | Perola | | | THL Biobank / Finnish Institute for Health and Welfare (THL), Helsinki, Finland | Steering Committee | University of Helsinki & Biobanks |
| Jukka | Partanen | | | Finnish Red Cross Blood Service / Finnish Hematology Registry and Clinical Biobank, Helsinki, Finland | Steering Committee | University of Helsinki & Biobanks |

*Indicates required information. Only first name, last name, and suffix will appear in PubMed.

| First Name and Middle Initial(s)* | Last Name* | Suffix* | Academic Degrees | Institution and location (city, state/province, country) | Role or Contribution, eg, chair, principal investigator | Subgroup, eg, Steering Committee |
|---|---|---|---|---|---|---|
| Taneli | Raivio | | | Helsinki Biobank / Helsinki University and Hospital District of Helsinki and Uusimaa, Helsinki | Steering Committee | University of Helsinki & Biobanks |
| Eero | Punkka | | | Helsinki Biobank / Helsinki University and Hospital District of Helsinki and Uusimaa, Helsinki | Steering Committee | University of Helsinki & Biobanks |
| Teija | Kekonen | | | Northern Finland Biobank Borealis / University of Oulu / Wellbeing services county of North Ostrobothnia, Oulu, Finland | Steering Committee | University of Helsinki & Biobanks |
| Raisa | Serpi | | | Northern Finland Biobank Borealis / University of Oulu / Wellbeing services county of North Ostrobothnia, Oulu, Finland | Steering Committee | University of Helsinki & Biobanks |
| Kati | Kristiansson | | | Finnish Clinical Biobank Tampere / University of Tampere / Wellbeing Services County of Pirkanmaa, Tampere, Finland | Steering Committee | University of Helsinki & Biobanks |

Supplemental Online Content: Nonauthor Collaborators

*Indicates required information. Only first name, last name, and suffix will appear in PubMed.

| First Name and Middle Initial(s)* | Last Name* | Suffix* | Academic Degrees | Institution and location (city, state/province, country) | Role or Contribution, eg, chair, principal investigator | Subgroup, eg, Steering Committee |
|---|---|---|---|---|---|---|
| Sanna | Siltanen | | | Finnish Clinical Biobank Tampere / University of Tampere / Wellbeing Services County of Pirkanmaa, Tampere, Finland | Steering Committee | University of Helsinki & Biobanks |
| Veli-Matti | Kosma | | | Biobank of Eastern Finland / University of Eastern Finland / Wellbeing services county of North Savo, Kuopio, Finland | Steering Committee | University of Helsinki & Biobanks |
| Arto | Mannermaa | | | Biobank of Eastern Finland / University of Eastern Finland / Wellbeing services county of North Savo, Kuopio, Finland | Steering Committee | University of Helsinki & Biobanks |
| Jari | Laukkanen | | | Central Finland Biobank / University of Jyväskylä / Wellbeing Services County of Central Finland, Jyväskylä, Finland | Steering Committee | University of Helsinki & Biobanks |
| Tiina | Jokela | | | Central Finland Biobank / University of Jyväskylä / Wellbeing Services County of Central Finland, Jyväskylä, Finland | Steering Committee | University of Helsinki & Biobanks |

*Indicates required information. Only first name, last name, and suffix will appear in PubMed.

| First Name and Middle Initial(s)* | Last Name* | Suffix* | Academic Degrees | Institution and location (city, state/province, country) | Role or Contribution, eg, chair, principal investigator | Subgroup, eg, Steering Committee |
|---|---|---|---|---|---|---|
| Mervi | Ahlroth | | | Finnish Biobank Cooperative - FINBB | Steering Committee | University of Helsinki & Biobanks |
| Johanna | Mäkelä | | | Finnish Biobank Cooperative - FINBB | Steering Committee | University of Helsinki & Biobanks |
| Outi | Tuovila | | | Business Finland, Helsinki, Finland | Steering Committee | Other Experts/ Non-Voting Members |
| Jeffrey | Waring | | | Abbvie, Chicago, IL, United States | Scientific Committee | Pharmaceutical companies |
| Bridget | Riley-Gillis | | | Abbvie, Chicago, IL, United States | Scientific Committee | Pharmaceutical companies |
| Fedik | Rahimov | | | Abbvie, Chicago, IL, United States | Scientific Committee | Pharmaceutical companies |
| Ioanna | Tachmazidou | | | Astra Zeneca, Cambridge, United Kingdom | Scientific Committee | Pharmaceutical companies |
| Slavé | Petrovski | | | Astra Zeneca, Cambridge, United Kingdom | Scientific Committee | Pharmaceutical companies |
| Alix | Berton | | | Bayer AG, Leverkusen, Germany | Scientific Committee | Pharmaceutical companies |
| Santha | Ramakrishnan | | | Bayer AG, Leverkusen, Germany | Scientific Committee | Pharmaceutical companies |
| Ellen | Tsai | | | Biogen, Cambridge, MA, United States | Scientific Committee | Pharmaceutical companies |
| Zhihao | Ding | | | Boehringer Ingelheim, Ingelheim am Rhein, Germany | Scientific Committee | Pharmaceutical companies |
| Marc | Jung | | | Boehringer Ingelheim, Ingelheim am Rhein, Germany | Scientific Committee | Pharmaceutical companies |

Supplemental Online Content: Nonauthor Collaborators

*Indicates required information. Only first name, last name, and suffix will appear in PubMed.

| First Name and Middle Initial(s)* | Last Name* | Suffix* | Academic Degrees | Institution and location (city, state/province, country) | Role or Contribution, eg, chair, principal investigator | Subgroup, eg, Steering Committee |
|---|---|---|---|---|---|---|
| Hanati | Tuoken | | | Boehringer Ingelheim, Ingelheim am Rhein, Germany | Scientific Committee | Pharmaceutical companies |
| Shameek | Biswas | | | Bristol Myers Squibb, New York, NY, United States | Scientific Committee | Pharmaceutical companies |
| Benjamin | Sun | | | Bristol Myers Squibb, New York, NY, United States | Scientific Committee | Pharmaceutical companies |
| Rion | Pendergrass | | | Genentech, San Francisco, CA, United States | Scientific Committee | Pharmaceutical companies |
| Jonathan | Davitte | | | GlaxoSmithKline, Collegeville, PA, United States | Scientific Committee | Pharmaceutical companies |
| Neha | Raghavan | | | Merck, Kenilworth, NJ, United States | Scientific Committee | Pharmaceutical companies |
| Jae-Hoon | Sul | | | Merck, Kenilworth, NJ, United States | Scientific Committee | Pharmaceutical companies |
| Melis Atalar | Aksit | | | Pfizer, New York, NY, United States | Scientific Committee | Pharmaceutical companies |
| Xinli | Hu | | | Pfizer, New York, NY, United States | Scientific Committee | Pharmaceutical companies |
| Katherine | Klinger | | | Translational Sciences, Sanofi R&D, Framingham, MA, USA | Scientific Committee | Pharmaceutical companies |
| Robert | Graham | | | Maze Therapeutics, San Francisco, CA, United States | Scientific Committee | Pharmaceutical companies |

Supplemental Online Content: Nonauthor Collaborators

*Indicates required information. Only first name, last name, and suffix will appear in PubMed.

| First Name and Middle Initial(s)* | Last Name* | Suffix* | Academic Degrees | Institution and location (city, state/province, country) | Role or Contribution, eg, chair, principal investigator | Subgroup, eg, Steering Committee |
|---|---|---|---|---|---|---|
| Dawn | Waterworth | | | Johnson & Johnson Innovative Medicine, Spring House, PA, United States | Scientific Committee | Pharmaceutical companies |
| Nicole | Renaud | | | Novartis Institutes for BioMedical Research, Cambridge, MA, United States | Scientific Committee | Pharmaceutical companies |
| Ma´en | Obeidat | | | Novartis Institutes for BioMedical Research, Cambridge, MA, United States | Scientific Committee | Pharmaceutical companies |
| Jonathan | Chung | | | Novartis Institutes for BioMedical Research, Cambridge, MA, United States | Scientific Committee | Pharmaceutical companies |
| Jonas | Zierer | | | Novartis Institutes for BioMedical Research, Cambridge, MA, United States | Scientific Committee | Pharmaceutical companies |
| Mari | Niemi | | | Novartis Institutes for BioMedical Research, Cambridge, MA, United States | Scientific Committee | Pharmaceutical companies |
| Samuli | Ripatti | | | Institute for Molecular Medicine Finland (FIMM), HiLIFE, University of Helsinki, Helsinki, Finland | Scientific Committee | University of Helsinki & Biobanks |

*Indicates required information. Only first name, last name, and suffix will appear in PubMed.

| First Name and Middle Initial(s)* | Last Name* | Suffix* | Academic Degrees | Institution and location (city, state/province, country) | Role or Contribution, eg, chair, principal investigator | Subgroup, eg, Steering Committee |
|---|---|---|---|---|---|---|
| Johanna | Schleutker | | | Auria Biobank / University of Turku / Wellbeing Services County of Southwest Finland, Turku, Finland | Scientific Committee | University of Helsinki & Biobanks |
| Markus | Perola | | | THL Biobank / Finnish Institute for Health and Welfare (THL), Helsinki, Finland | Scientific Committee | University of Helsinki & Biobanks |
| Tiina | Wahlfors | | | THL Biobank / Finnish Institute for Health and Welfare (THL), Helsinki, Finland | Scientific Committee | University of Helsinki & Biobanks |
| Mikko | Arvas | | | Finnish Red Cross Blood Service / Finnish Hematology Registry and Clinical Biobank, Helsinki, Finland | Scientific Committee | University of Helsinki & Biobanks |
| Olli | Carpén | | | Helsinki Biobank / Helsinki University and Hospital District of Helsinki and Uusimaa, Helsinki | Scientific Committee | University of Helsinki & Biobanks |
| Reetta | Hinttala | | | Northern Finland Biobank Borealis / University of Oulu / Wellbeing services county of North Ostrobothnia, Oulu, Finland | Scientific Committee | University of Helsinki & Biobanks |

*Indicates required information. Only first name, last name, and suffix will appear in PubMed.

| First Name and Middle Initial(s)* | Last Name* | Suffix* | Academic Degrees | Institution and location (city, state/province, country) | Role or Contribution, eg, chair, principal investigator | Subgroup, eg, Steering Committee |
|---|---|---|---|---|---|---|
| Johannes | Kettunen | | | Northern Finland Biobank Borealis / University of Oulu / Wellbeing services county of North Ostrobothnia, Oulu, Finland | Scientific Committee | University of Helsinki & Biobanks |
| Arto | Mannermaa | | | Biobank of Eastern Finland / University of Eastern Finland / Wellbeing services county of North Savo, Kuopio, Finland | Scientific Committee | University of Helsinki & Biobanks |
| Katriina | Aalto-Setälä | | | Faculty of Medicine and Health Technology, Tampere University, Tampere, Finland | Scientific Committee | University of Helsinki & Biobanks |
| Mika | Kähönen | | | Finnish Clinical Biobank Tampere / University of Tampere / Wellbeing Services County of Pirkanmaa, Tampere, Finland | Scientific Committee | University of Helsinki & Biobanks |
| Jari | Laukkanen | | | Central Finland Biobank / University of Jyväskylä / Wellbeing Services County of Central Finland, Jyväskylä, Finland | Scientific Committee | University of Helsinki & Biobanks |
| Johanna | Mäkelä | | | FINBB - Finnish biobank cooperative | Scientific Committee | University of Helsinki & Biobanks |

Supplemental Online Content: Nonauthor Collaborators

*Indicates required information. Only first name, last name, and suffix will appear in PubMed.

| First Name and Middle Initial(s)* | Last Name* | Suffix* | Academic Degrees | Institution and location (city, state/province, country) | Role or Contribution, eg, chair, principal investigator | Subgroup, eg, Steering Committee |
|---|---|---|---|---|---|---|
| Hanna | Kujala | | | Biobank of Eastern Finland / University of Eastern Finland / Wellbeing services county of North Savo, Kuopio, Finland | Clinical Group / Task Force | |
| Triin | Laisk | | | Estonian biobank, Tartu, Estonia | Clinical Group / Task Force | |
| Natalia | Pujol | | | Estonian biobank, Tartu, Estonia | Clinical Group / Task Force | |
| Mika | Kähönen | | | Finnish Clinical Biobank Tampere / University of Tampere / Wellbeing Services County of Pirkanmaa, Tampere, Finland | Clinical Group / Task Force | |
| Veikko | Salomaa | | | Finnish Institute for Health and Welfare (THL), Helsinki, Finland | Clinical Group / Task Force | |
| Jaana | Suvisaari | | | Finnish Institute for Health and Welfare (THL), Helsinki, Finland | Clinical Group / Task Force | |
| Satu | Koskela | | | Finnish Red Cross Blood Service / Finnish Hematology Registry and Clinical Biobank, Helsinki, Finland | Clinical Group / Task Force | |

Supplemental Online Content: Nonauthor Collaborators

*Indicates required information. Only first name, last name, and suffix will appear in PubMed.

| First Name and Middle Initial(s)* | Last Name* | Suffix* | Academic Degrees | Institution and location (city, state/province, country) | Role or Contribution, eg, chair, principal investigator | Subgroup, eg, Steering Committee |
|---|---|---|---|---|---|---|
| Jouni | Lauronen | | | Finnish Red Cross Blood Service / Finnish Hematology Registry and Clinical Biobank, Helsinki, Finland | Clinical Group / Task Force | |
| Kristiina | Aittomäki | | | Helsinki University Central Hospital, Helsinki, Finland | Clinical Group / Task Force | |
| Pirkko | Pussinen | | | Helsinki University Hospital and University of Helsinki, Helsinki / University of Eastern Finland. Kuopio, Finland | Clinical Group / Task Force | |
| Tuomo | Meretoja | | | Helsinki University Hospital and University of Helsinki, Helsinki, Finland | Clinical Group / Task Force | |
| Heikki | Joensuu | | | Helsinki University Hospital and University of Helsinki, Helsinki, Finland | Clinical Group / Task Force | |
| Peeter | Karihtala | | | Helsinki University Hospital and University of Helsinki, Helsinki, Finland | Clinical Group / Task Force | |
| Emma | Juuri | | | Helsinki University Hospital and University of Helsinki, Helsinki, Finland | Clinical Group / Task Force | |

Supplemental Online Content: Nonauthor Collaborators

*Indicates required information. Only first name, last name, and suffix will appear in PubMed.

| First Name and Middle Initial(s)* | Last Name* | Suffix* | Academic Degrees | Institution and location (city, state/province, country) | Role or Contribution, eg, chair, principal investigator | Subgroup, eg, Steering Committee |
|---|---|---|---|---|---|---|
| Aino | Salminen | | | Helsinki University Hospital and University of Helsinki, Helsinki, Finland | Clinical Group / Task Force | |
| Tuula | Salo | | | Helsinki University Hospital and University of Helsinki, Helsinki, Finland | Clinical Group / Task Force | |
| David | Rice | | | Helsinki University Hospital and University of Helsinki, Helsinki, Finland | Clinical Group / Task Force | |
| Pekka | Nieminen | | | Helsinki University Hospital and University of Helsinki, Helsinki, Finland | Clinical Group / Task Force | |
| Ulla | Palotie | | | Helsinki University Hospital and University of Helsinki, Helsinki, Finland | Clinical Group / Task Force | |
| Fredrik | Åberg | | | Helsinki University Hospital and University of Helsinki, Helsinki, Finland | Clinical Group / Task Force | |
| Daniel | Gordin | | | Helsinki University Hospital and University of Helsinki, Helsinki, Finland | Clinical Group / Task Force | |

Supplemental Online Content: Nonauthor Collaborators

*Indicates required information. Only first name, last name, and suffix will appear in PubMed.

| First Name and Middle Initial(s)* | Last Name* | Suffix* | Academic Degrees | Institution and location (city, state/province, country) | Role or Contribution, eg, chair, principal investigator | Subgroup, eg, Steering Committee |
|---|---|---|---|---|---|---|
| Patrik | Finne | | | Helsinki University Hospital and University of Helsinki, Helsinki, Finland | Clinical Group / Task Force | |
| Joni A | Turunen | | | Helsinki University Hospital and University of Helsinki, Helsinki, Finland; Folkhälsan Research Center, Helsinki, Finland | Clinical Group / Task Force | |
| Minna | Raivio | | | Hospital District of Helsinki and Uusimaa, Helsinki, Finland | Clinical Group / Task Force | |
| Pentti | Tienari | | | Hospital District of Helsinki and Uusimaa, Helsinki, Finland | Clinical Group / Task Force | |
| Martti | Färkkilä | | | Hospital District of Helsinki and Uusimaa, Helsinki, Finland | Clinical Group / Task Force | |
| Jukka | Koskela | | | Hospital District of Helsinki and Uusimaa, Helsinki, Finland | Clinical Group / Task Force | |
| Sampsa | Pikkarainen | | | Hospital District of Helsinki and Uusimaa, Helsinki, Finland | Clinical Group / Task Force | |
| Kari | Eklund | | | Hospital District of Helsinki and Uusimaa, Helsinki, Finland | Clinical Group / Task Force | |
| Paula | Kauppi | | | Hospital District of Helsinki and Uusimaa, Helsinki, Finland | Clinical Group / Task Force | |

Supplemental Online Content: Nonauthor Collaborators

*Indicates required information. Only first name, last name, and suffix will appear in PubMed.

| First Name and Middle Initial(s)* | Last Name* | Suffix* | Academic Degrees | Institution and location (city, state/province, country) | Role or Contribution, eg, chair, principal investigator | Subgroup, eg, Steering Committee |
|---|---|---|---|---|---|---|
| Daniel | Gordin | | | Hospital District of Helsinki and Uusimaa, Helsinki, Finland | Clinical Group / Task Force | |
| Juha | Sinisalo | | | Hospital District of Helsinki and Uusimaa, Helsinki, Finland | Clinical Group / Task Force | |
| Marja-Riitta | Taskinen | | | Hospital District of Helsinki and Uusimaa, Helsinki, Finland | Clinical Group / Task Force | |
| Tiinamaija | Tuomi | | | Hospital District of Helsinki and Uusimaa, Helsinki, Finland | Clinical Group / Task Force | |
| Timo | Hiltunen | | | Hospital District of Helsinki and Uusimaa, Helsinki, Finland | Clinical Group / Task Force | |
| Johanna | Mattson | | | Hospital District of Helsinki and Uusimaa, Helsinki, Finland | Clinical Group / Task Force | |
| Eveliina | Salminen | | | Hospital District of Helsinki and Uusimaa, Helsinki, Finland | Clinical Group / Task Force | |
| Terhi | Ollila | | | Hospital District of Helsinki and Uusimaa, Helsinki, Finland | Clinical Group / Task Force | |
| Katariina | Hannula-Jouppi | | | Hospital District of Helsinki and Uusimaa, Helsinki, Finland | Clinical Group / Task Force | |
| Oskari | Heikinheimo | | | Hospital District of Helsinki and Uusimaa, Helsinki, Finland | Clinical Group / Task Force | |

Supplemental Online Content: Nonauthor Collaborators

*Indicates required information. Only first name, last name, and suffix will appear in PubMed.

| First Name and Middle Initial(s)* | Last Name* | Suffix* | Academic Degrees | Institution and location (city, state/province, country) | Role or Contribution, eg, chair, principal investigator | Subgroup, eg, Steering Committee |
|---|---|---|---|---|---|---|
| Ilkka | Kalliala | | | Hospital District of Helsinki and Uusimaa, Helsinki, Finland | Clinical Group / Task Force | |
| Lauri | Aaltonen | | | Hospital District of Helsinki and Uusimaa, Helsinki, Finland | Clinical Group / Task Force | |
| Erkki | Isometsä | | | Hospital District of Helsinki and Uusimaa, Helsinki, Finland | Clinical Group / Task Force | |
| Antti | Aarnisalo | | | Hospital District of Helsinki and Uusimaa, Helsinki, Finland | Clinical Group / Task Force | |
| Ilkka | Immonen | | | Hospital District of Helsinki and Uusimaa, Helsinki, Finland | Clinical Group / Task Force | |
| Salla | Ranta | | | Hospital District of Helsinki and Uusimaa, Helsinki, Finland | Clinical Group / Task Force | |
| Filip | Scheperjans | | | Hospital District of Helsinki and Uusimaa, Helsinki, Finland | Clinical Group / Task Force | |
| Felix | Vaura | | | Institute for Molecular Medicine Finland (FIMM), HiLIFE, University of Helsinki, Helsinki, Finland | Clinical Group / Task Force | |
| Nina | Mars | | | Institute for Molecular Medicine Finland (FIMM), HiLIFE, University of Helsinki, Helsinki, Finland | Clinical Group / Task Force | |

Supplemental Online Content: Nonauthor Collaborators

*Indicates required information. Only first name, last name, and suffix will appear in PubMed.

| First Name and Middle Initial(s)* | Last Name* | Suffix* | Academic Degrees | Institution and location (city, state/province, country) | Role or Contribution, eg, chair, principal investigator | Subgroup, eg, Steering Committee |
|---|---|---|---|---|---|---|
| Esa | Pitkänen | | | Institute for Molecular Medicine Finland (FIMM), HiLIFE, University of Helsinki, Helsinki, Finland | Clinical Group / Task Force | |
| Hannele | Laivuori | | | Institute for Molecular Medicine Finland (FIMM), HiLIFE, University of Helsinki, Helsinki, Finland | Clinical Group / Task Force | |
| Katja | Kivinen | | | Institute for Molecular Medicine Finland (FIMM), HiLIFE, University of Helsinki, Helsinki, Finland | Clinical Group / Task Force | |
| Elisabeth | Widen | | | Institute for Molecular Medicine Finland (FIMM), HiLIFE, University of Helsinki, Helsinki, Finland | Clinical Group / Task Force | |
| Taru | Tukiainen | | | Institute for Molecular Medicine Finland (FIMM), HiLIFE, University of Helsinki, Helsinki, Finland | Clinical Group / Task Force | |
| Hanna | Ollila | | | Institute for Molecular Medicine Finland (FIMM), HiLIFE, University of Helsinki, Helsinki, Finland | Clinical Group / Task Force | |

Supplemental Online Content: Nonauthor Collaborators

*Indicates required information. Only first name, last name, and suffix will appear in PubMed.

| First Name and Middle Initial(s)* | Last Name* | Suffix* | Academic Degrees | Institution and location (city, state/province, country) | Role or Contribution, eg, chair, principal investigator | Subgroup, eg, Steering Committee |
|---|---|---|---|---|---|---|
| Elmo | Saarentaus | | | Institute for Molecular Medicine Finland (FIMM), HiLIFE, University of Helsinki, Helsinki, Finland | Clinical Group / Task Force | |
| Anne | Kerola | | | Institute for Molecular Medicine Finland (FIMM), HiLIFE, University of Helsinki, Helsinki, Finland | Clinical Group / Task Force | |
| Eero | Vuoksimaa | | | Institute for Molecular Medicine Finland (FIMM), HiLIFE, University of Helsinki, Helsinki, Finland | Clinical Group / Task Force | |
| Joni | Lindbohm | | | Institute for Molecular Medicine Finland (FIMM), HiLIFE, University of Helsinki, Helsinki, Finland | Clinical Group / Task Force | |
| Zhiyu | Yang | | | Institute for Molecular Medicine Finland (FIMM), HiLIFE, University of Helsinki, Helsinki, Finland | Clinical Group / Task Force | |

Supplemental Online Content: Nonauthor Collaborators

*Indicates required information. Only first name, last name, and suffix will appear in PubMed.

| First Name and Middle Initial(s)* | Last Name* | Suffix* | Academic Degrees | Institution and location (city, state/province, country) | Role or Contribution, eg, chair, principal investigator | Subgroup, eg, Steering Committee |
|---|---|---|---|---|---|---|
| Matthew | Sampson | | | Institute for Molecular Medicine Finland (FIMM), HiLIFE, University of Helsinki, Helsinki, Finland; Broad Institute & Harvard Medical School, Cambridge, United States | Clinical Group / Task Force | |
| Adrian | Banerji | | | Institute for Molecular Medicine Finland (FIMM), HiLIFE, University of Helsinki, Helsinki, Finland; Broad Institute & Harvard Medical School, Cambridge, United States | Clinical Group / Task Force | |
| Michelle | McNulty | | | Institute for Molecular Medicine Finland (FIMM), HiLIFE, University of Helsinki, Helsinki, Finland; Broad Institute & Harvard Medical School, Cambridge, United States | Clinical Group / Task Force | |

*Indicates required information. Only first name, last name, and suffix will appear in PubMed.

| First Name and Middle Initial(s)* | Last Name* | Suffix* | Academic Degrees | Institution and location (city, state/province, country) | Role or Contribution, eg, chair, principal investigator | Subgroup, eg, Steering Committee |
|---|---|---|---|---|---|---|
| Aoxing | Liu | | | Institute for Molecular Medicine Finland (FIMM), HiLIFE, University of Helsinki, Helsinki, Finland; Broad Institute, Cambridge, MA, United States | Clinical Group / Task Force | |
| Joel | Rämö | | | Institute for Molecular Medicine Finland (FIMM), HiLIFE, University of Helsinki, Helsinki, Finland; Broad Institute, Cambridge, MA, United States | Clinical Group / Task Force | |
| Austin | Argentieri | | | Institute for Molecular Medicine Finland (FIMM), HiLIFE, University of Helsinki, Helsinki, Finland; Broad Institute, Cambridge, MA, United States | Clinical Group / Task Force | |
| Amanda | Elliott | | | Institute for Molecular Medicine Finland (FIMM), HiLIFE, University of Helsinki, Helsinki, Finland; Broad Institute, Cambridge, MA, USA and Massachusetts General Hospital, Boston, MA, USA | Clinical Group / Task Force | |

Supplemental Online Content: Nonauthor Collaborators

*Indicates required information. Only first name, last name, and suffix will appear in PubMed.

| First Name and Middle Initial(s)* | Last Name* | Suffix* | Academic Degrees | Institution and location (city, state/province, country) | Role or Contribution, eg, chair, principal investigator | Subgroup, eg, Steering Committee |
|---|---|---|---|---|---|---|
| Elisa | Rahikkala | | | Northern Ostrobothnia Hospital District, Oulu, Finland | Clinical Group / Task Force | |
| Kirsi | Sipilä | | | Oulu University Hospital and University of Oulu, Oulu, Finland | Clinical Group / Task Force | |
| Valtteri | Julkunen | | | University of Eastern Finland and Kuopio University Hospital, Kuopio, Finland | Clinical Group / Task Force | |
| Ville | Leinonen | | | University of Eastern Finland and Kuopio University Hospital, Kuopio, Finland | Clinical Group / Task Force | |
| Sanna | Toppila-Salmi | | | University of Eastern Finland and Kuopio University Hospital, Kuopio, Finland; Helsinki University Hospital and University of Helsinki, Finland | Clinical Group / Task Force | |
| Mikko | Hiltunen | | | University of Eastern Finland, Kuopio, Finland | Clinical Group / Task Force | |
| Eino | Solje | | | University of Eastern Finland, Kuopio, Finland | Clinical Group / Task Force | |

Supplemental Online Content: Nonauthor Collaborators

*Indicates required information. Only first name, last name, and suffix will appear in PubMed.

| First Name and Middle Initial(s)* | Last Name* | Suffix* | Academic Degrees | Institution and location (city, state/province, country) | Role or Contribution, eg, chair, principal investigator | Subgroup, eg, Steering Committee |
|---|---|---|---|---|---|---|
| Hannu | Kankaanranta | | | University of Gothenburg, Gothenburg, Sweden/ Seinäjoki Central Hospital, Seinäjoki, Finland/ Tampere University, Tampere, Finland | Clinical Group / Task Force | |
| Antti | Mäkitie | | | University of Helsinki and Helsinki University Hospital, Helsinki, Finland | Clinical Group / Task Force | |
| Iiris | Hovatta | | | University of Helsinki, Helsinki, Finland | Clinical Group / Task Force | |
| Niko | Välimäki | | | University of Helsinki, Helsinki, Finland | Clinical Group / Task Force | |
| Minttu | Marttila | | | University of Helsinki, Helsinki, Finland | Clinical Group / Task Force | |
| Anne | Portaankorva | | | University of Helsinki, Helsinki, Finland | Clinical Group / Task Force | |
| Eija | Laakkonen | | | University of Jyväskylä, Jyväskylä, Finland | Clinical Group / Task Force | |
| Heidi | Silven | | | University of Oulu, Oulu, Finland | Clinical Group / Task Force | |
| Eeva | Sliz | | | University of Oulu, Oulu, Finland | Clinical Group / Task Force | |
| Riikka | Arffman | | | University of Oulu, Oulu, Finland | Clinical Group / Task Force | |
| Susanna | Savukoski | | | University of Oulu, Oulu, Finland | Clinical Group / Task Force | |

Supplemental Online Content: Nonauthor Collaborators

*Indicates required information. Only first name, last name, and suffix will appear in PubMed.

| First Name and Middle Initial(s)* | Last Name* | Suffix* | Academic Degrees | Institution and location (city, state/province, country) | Role or Contribution, eg, chair, principal investigator | Subgroup, eg, Steering Committee |
|---|---|---|---|---|---|---|
| Riitta | Kaarteenaho | | | University of Oulu, Oulu, Finland | Clinical Group / Task Force | |
| Jaakko | Tyrmi | | | University of Oulu, Oulu, Finland / University of Tampere, Tampere, Finland | Clinical Group / Task Force | |
| Laura | Kuusalo | | | University of Turku, Turku, Finland | Clinical Group / Task Force | |
| Laura | Pirilä | | | University of Turku, Turku, Finland | Clinical Group / Task Force | |
| Tapio | Hellman | | | University of Turku, Turku, Finland | Clinical Group / Task Force | |
| Matti | Vuori | | | University of Turku, Turku, Finland | Clinical Group / Task Force | |
| Teemu | Niiranen | | | University of Turku, Turku, Finland; Finnish Institute for Health and Welfare (THL), Helsinki, Finland | Clinical Group / Task Force | |
| Timo | Blomster | | | Wellbeing services county of North Ostrobothnia, Oulu, Finland | Clinical Group / Task Force | |
| Johanna | Huhtakangas | | | Wellbeing services county of North Ostrobothnia, Oulu, Finland | Clinical Group / Task Force | |
| Terttu | Harju | | | Wellbeing services county of North Ostrobothnia, Oulu, Finland | Clinical Group / Task Force | |

Supplemental Online Content: Nonauthor Collaborators

*Indicates required information. Only first name, last name, and suffix will appear in PubMed.

| First Name and Middle Initial(s)* | Last Name* | Suffix* | Academic Degrees | Institution and location (city, state/province, country) | Role or Contribution, eg, chair, principal investigator | Subgroup, eg, Steering Committee |
|---|---|---|---|---|---|---|
| Kaisa | Tasanen | | | Wellbeing services county of North Ostrobothnia, Oulu, Finland | Clinical Group / Task Force | |
| Laura | Huilaja | | | Wellbeing services county of North Ostrobothnia, Oulu, Finland | Clinical Group / Task Force | |
| Vuokko | Anttonen | | | Wellbeing services county of North Ostrobothnia, Oulu, Finland | Clinical Group / Task Force | |
| Marja | Vääräsmäki | | | Wellbeing services county of North Ostrobothnia, Oulu, Finland | Clinical Group / Task Force | |
| Outi | Uimari | | | Wellbeing services county of North Ostrobothnia, Oulu, Finland | Clinical Group / Task Force | |
| Laure | Morin-Papunen | | | Wellbeing services county of North Ostrobothnia, Oulu, Finland | Clinical Group / Task Force | |
| Maarit | Niinimäki | | | Wellbeing services county of North Ostrobothnia, Oulu, Finland | Clinical Group / Task Force | |

*Indicates required information. Only first name, last name, and suffix will appear in PubMed.

| First Name and Middle Initial(s)* | Last Name* | Suffix* | Academic Degrees | Institution and location (city, state/province, country) | Role or Contribution, eg, chair, principal investigator | Subgroup, eg, Steering Committee |
|---|---|---|---|---|---|---|
| Terhi | Piltonen | | | Wellbeing services county of North Ostrobothnia, Oulu, Finland | Clinical Group / Task Force | |
| Reetta | Kälviäinen | | | Wellbeing services county of North Savo, Kuopio, Finland | Clinical Group / Task Force | |
| Valtteri | Julkunen | | | Wellbeing services county of North Savo, Kuopio, Finland | Clinical Group / Task Force | |
| Hilkka | Soininen | | | Wellbeing services county of North Savo, Kuopio, Finland | Clinical Group / Task Force | |
| Mikko | Kiviniemi | | | Wellbeing services county of North Savo, Kuopio, Finland | Clinical Group / Task Force | |
| Oili | Kaipiainen-Seppänen | | | Wellbeing services county of North Savo, Kuopio, Finland | Clinical Group / Task Force | |
| Margit | Pelkonen | | | Wellbeing services county of North Savo, Kuopio, Finland | Clinical Group / Task Force | |
| Päivi | Auvinen | | | Wellbeing services county of North Savo, Kuopio, Finland | Clinical Group / Task Force | |
| Maria | Siponen | | | Wellbeing services county of North Savo, Kuopio, Finland | Clinical Group / Task Force | |
| Liisa | Suominen | | | Wellbeing services county of North Savo, Kuopio, Finland | Clinical Group / Task Force | |

Supplemental Online Content: Nonauthor Collaborators

*Indicates required information. Only first name, last name, and suffix will appear in PubMed.

| First Name and Middle Initial(s)* | Last Name* | Suffix* | Academic Degrees | Institution and location (city, state/province, country) | Role or Contribution, eg, chair, principal investigator | Subgroup, eg, Steering Committee |
|---|---|---|---|---|---|---|
| Päivi | Mäntylä | | | Wellbeing services county of North Savo, Kuopio, Finland | Clinical Group / Task Force | |
| Kai | Kaarniranta | | | Wellbeing services county of North Savo, Kuopio, Finland; University of Lodz, Lodz, Poland | Clinical Group / Task Force | |
| Jukka | Peltola | | | Wellbeing Services County of Pirkanmaa, Tampere, Finland | Clinical Group / Task Force | |
| Airi | Jussila | | | Wellbeing Services County of Pirkanmaa, Tampere, Finland | Clinical Group / Task Force | |
| Katri | Kaukinen | | | Wellbeing Services County of Pirkanmaa, Tampere, Finland | Clinical Group / Task Force | |
| Pia | Isomäki | | | Wellbeing Services County of Pirkanmaa, Tampere, Finland | Clinical Group / Task Force | |
| Jussi | Hernesniemi | | | Wellbeing Services County of Pirkanmaa, Tampere, Finland | Clinical Group / Task Force | |
| Annika | Auranen | | | Wellbeing Services County of Pirkanmaa, Tampere, Finland | Clinical Group / Task Force | |
| Hannu | Uusitalo | | | Wellbeing Services County of Pirkanmaa, Tampere, Finland | Clinical Group / Task Force | |

Supplemental Online Content: Nonauthor Collaborators

*Indicates required information. Only first name, last name, and suffix will appear in PubMed.

| First Name and Middle Initial(s)* | Last Name* | Suffix* | Academic Degrees | Institution and location (city, state/province, country) | Role or Contribution, eg, chair, principal investigator | Subgroup, eg, Steering Committee |
|---|---|---|---|---|---|---|
| Teea | Salmi | | | Wellbeing Services County of Pirkanmaa, Tampere, Finland | Clinical Group / Task Force | |
| Venla | Kurra | | | Wellbeing Services County of Pirkanmaa, Tampere, Finland | Clinical Group / Task Force | |
| Laura | Kotaniemi-Talonen | | | Wellbeing Services County of Pirkanmaa, Tampere, Finland | Clinical Group / Task Force | |
| Argyro | Bizaki-Vallaskangas | | | Wellbeing Services County of Pirkanmaa, Tampere, Finland | Clinical Group / Task Force | |
| Juha | Rinne | | | Wellbeing Services County of Southwest Finland, Turku, Finland | Clinical Group / Task Force | |
| Roosa | Kallionpää | | | Wellbeing Services County of Southwest Finland, Turku, Finland | Clinical Group / Task Force | |
| Markku | Voutilainen | | | Wellbeing Services County of Southwest Finland, Turku, Finland | Clinical Group / Task Force | |
| Antti | Palomäki | | | Wellbeing Services County of Southwest Finland, Turku, Finland | Clinical Group / Task Force | |
| Laura | Pirilä | | | Wellbeing Services County of Southwest Finland, Turku, Finland | Clinical Group / Task Force | |
| Riitta | Lahesmaa | | | Wellbeing Services County of Southwest Finland, Turku, Finland | Clinical Group / Task Force | |

Supplemental Online Content: Nonauthor Collaborators

*Indicates required information. Only first name, last name, and suffix will appear in PubMed.

| First Name and Middle Initial(s)* | Last Name* | Suffix* | Academic Degrees | Institution and location (city, state/province, country) | Role or Contribution, eg, chair, principal investigator | Subgroup, eg, Steering Committee |
|---|---|---|---|---|---|---|
| Kaj | Metsärinne | | | Wellbeing Services County of Southwest Finland, Turku, Finland | Clinical Group / Task Force | |
| Jenni | Aittokallio | | | Wellbeing Services County of Southwest Finland, Turku, Finland | Clinical Group / Task Force | |
| Klaus | Elenius | | | Wellbeing Services County of Southwest Finland, Turku, Finland | Clinical Group / Task Force | |
| Sirkku | Peltonen | | | Wellbeing Services County of Southwest Finland, Turku, Finland | Clinical Group / Task Force | |
| Leena | Koulu | | | Wellbeing Services County of Southwest Finland, Turku, Finland | Clinical Group / Task Force | |
| Ulvi | Gursoy | | | Wellbeing Services County of Southwest Finland, Turku, Finland | Clinical Group / Task Force | |
| Varpu | Jokimaa | | | Wellbeing Services County of Southwest Finland, Turku, Finland | Clinical Group / Task Force | |
| Tytti | Willberg | | | Wellbeing Services County of Southwest Finland, Turku, Finland | Clinical Group / Task Force | |
| Adam | Ziemann | | | Abbvie, Chicago, IL, United States | Clinical Group / Task Force | |
| Nizar | Smaoui | | | Abbvie, Chicago, IL, United States | Clinical Group / Task Force | |
| Anne | Lehtonen | | | Abbvie, Chicago, IL, United States | Clinical Group / Task Force | |

Supplemental Online Content: Nonauthor Collaborators

*Indicates required information. Only first name, last name, and suffix will appear in PubMed.

| First Name and Middle Initial(s)* | Last Name* | Suffix* | Academic Degrees | Institution and location (city, state/province, country) | Role or Contribution, eg, chair, principal investigator | Subgroup, eg, Steering Committee |
|---|---|---|---|---|---|---|
| Apinya | Lertratanakul | | | Abbvie, Chicago, IL, United States | Clinical Group / Task Force | |
| Relja | Popovic | | | Abbvie, Chicago, IL, United States | Clinical Group / Task Force | |
| Mengzhen | Liu | | | Abbvie, Chicago, IL, United States | Clinical Group / Task Force | |
| Anneke | Den Hollander | | | AbbVie, Chicago, IL, United States | Clinical Group / Task Force | |
| Jan | Freudenberg | | | AbbVie, Chicago, IL, United States | Clinical Group / Task Force | |
| Britney | Milkovich | | | AbbVie, Chicago, IL, United States | Clinical Group / Task Force | |
| Andrew | Blumenfeld | | | AbbVie, Chicago, IL, United States | Clinical Group / Task Force | |
| Tushar | Kumar | | | AbbVie, Chicago, IL, United States | Clinical Group / Task Force | |
| Dirk | Paul | | | Astra Zeneca, Cambridge, United Kingdom | Clinical Group / Task Force | |
| Bram | Prins | | | Astra Zeneca, Cambridge, United Kingdom | Clinical Group / Task Force | |
| Eleanor | Wheeler | | | Astra Zeneca, Cambridge, United Kingdom | Clinical Group / Task Force | |
| Kousik | Kundu | | | Astra Zeneca, Cambridge, United Kingdom | Clinical Group / Task Force | |
| Santosh | Atanur | | | Astra Zeneca, Cambridge, United Kingdom | Clinical Group / Task Force | |

Supplemental Online Content: Nonauthor Collaborators

*Indicates required information. Only first name, last name, and suffix will appear in PubMed.

| First Name and Middle Initial(s)* | Last Name* | Suffix* | Academic Degrees | Institution and location (city, state/province, country) | Role or Contribution, eg, chair, principal investigator | Subgroup, eg, Steering Committee |
|---|---|---|---|---|---|---|
| Andrew | Lowe | | | Astra Zeneca, Cambridge, United Kingdom | Clinical Group / Task Force | |
| Thomas | Spargo | | | Astra Zeneca, Cambridge, United Kingdom | Clinical Group / Task Force | |
| Oliver | Burren | | | Astra Zeneca, Cambridge, United Kingdom | Clinical Group / Task Force | |
| Margarete | Fabre | | | AstraZeneca, Cambridge, United Kingdom | Clinical Group / Task Force | |
| Fabio | Baschiera | | | Bayer AG, Leverkusen, Germany | Clinical Group / Task Force | |
| Hans | van Leeuwen | | | Bayer AG, Leverkusen, Germany | Clinical Group / Task Force | |
| Himanshu | Manchanda | | | Bayer AG, Leverkusen, Germany | Clinical Group / Task Force | |
| Karl | Heilbron | | | Bayer AG, Leverkusen, Germany | Clinical Group / Task Force | |
| Martin | Rao | | | Bayer AG, Leverkusen, Germany | Clinical Group / Task Force | |
| Nicole | Schmidt | | | Bayer AG, Leverkusen, Germany | Clinical Group / Task Force | |
| Samu | Kurki | | | Bayer AG, Leverkusen, Germany | Clinical Group / Task Force | |
| Johanna | Mielke | | | Bayer AG, Leverkusen, Germany | Clinical Group / Task Force | |
| Juho | Immonen | | | Bayer AG, Leverkusen, Germany | Clinical Group / Task Force | |
| Thomas | Battram | | | Bayer AG, Leverkusen, Germany | Clinical Group / Task Force | |

Supplemental Online Content: Nonauthor Collaborators

*Indicates required information. Only first name, last name, and suffix will appear in PubMed.

| First Name and Middle Initial(s)* | Last Name* | Suffix* | Academic Degrees | Institution and location (city, state/province, country) | Role or Contribution, eg, chair, principal investigator | Subgroup, eg, Steering Committee |
|---|---|---|---|---|---|---|
| Tobias | Hogrebe | | | Bayer AG, Leverkusen, Germany | Clinical Group / Task Force | |
| Susan | Eaton | | | Biogen, Cambridge, MA, United States | Clinical Group / Task Force | |
| Ketian | Yu | | | Biogen, Cambridge, MA, United States | Clinical Group / Task Force | |
| Stephanie | Loomis | | | Biogen, Cambridge, MA, United States | Clinical Group / Task Force | |
| Coro | Paisan-Ruiz | | | Biogen, Cambridge, MA, United States | Clinical Group / Task Force | |
| Elke | Markert | | | Boehringer Ingelheim, Ingelheim am Rhein, Germany | Clinical Group / Task Force | |
| Frank | Li | | | Boehringer Ingelheim, Ingelheim am Rhein, Germany | Clinical Group / Task Force | |
| Yao | Hu | | | Boehringer Ingelheim, Ingelheim am Rhein, Germany | Clinical Group / Task Force | |
| Christoph | Ogris | | | Boehringer Ingelheim, Ingelheim am Rhein, Germany | Clinical Group / Task Force | |
| Eric | Simon | | | Boehringer Ingelheim, Ingelheim am Rhein, Germany | Clinical Group / Task Force | |
| Julio Cesar | Bolivar Lopez | | | Boehringer Ingelheim, Ingelheim am Rhein, Germany | Clinical Group / Task Force | |
| Monika | Frysz | | | Boehringer Ingelheim, Ingelheim am Rhein, Germany | Clinical Group / Task Force | |

Supplemental Online Content: Nonauthor Collaborators

*Indicates required information. Only first name, last name, and suffix will appear in PubMed.

| First Name and Middle Initial(s)* | Last Name* | Suffix* | Academic Degrees | Institution and location (city, state/province, country) | Role or Contribution, eg, chair, principal investigator | Subgroup, eg, Steering Committee |
|---|---|---|---|---|---|---|
| Marla | Hochfeld | | | Bristol Myers Squibb, New York, NY, United States | Clinical Group / Task Force | |
| Cara | Carty | | | Bristol Myers Squibb, New York, NY, United States | Clinical Group / Task Force | |
| Michael | Turchin | | | Bristol Myers Squibb, New York, NY, United States | Clinical Group / Task Force | |
| Neelakshi | Jog | | | Bristol Myers Squibb, New York, NY, United States | Clinical Group / Task Force | |
| Corneliu | Bodea | | | Bristol Myers Squibb, New York, NY, United States | Clinical Group / Task Force | |
| Janie | Shelton | | | Bristol Myers Squibb, New York, NY, United States | Clinical Group / Task Force | |
| Chen | Li | | | Bristol Myers Squibb, New York, NY, United States | Clinical Group / Task Force | |
| Kritika | Singh | | | Bristol Myers Squibb, New York, NY, United States | Clinical Group / Task Force | |
| Peng | Jiang | | | Bristol Myers Squibb, New York, NY, United States | Clinical Group / Task Force | |
| Stephanie | Loomis | | | Bristol Myers Squibb, New York, NY, United States | Clinical Group / Task Force | |

Supplemental Online Content: Nonauthor Collaborators

*Indicates required information. Only first name, last name, and suffix will appear in PubMed.

| First Name and Middle Initial(s)* | Last Name* | Suffix* | Academic Degrees | Institution and location (city, state/province, country) | Role or Contribution, eg, chair, principal investigator | Subgroup, eg, Steering Committee |
|---|---|---|---|---|---|---|
| Elena | Sanchez | | | Bristol Myers Squibb, New York, NY, United States | Clinical Group / Task Force | |
| Lilith | Moss | | | Bristol Myers Squibb, New York, NY, United States | Clinical Group / Task Force | |
| Zijie | Zhao | | | Bristol Myers Squibb, New York, NY, United States | Clinical Group / Task Force | |
| Anna | Podgornaia | | | Bristol Myers Squibb, New York, NY, United States | Clinical Group / Task Force | |
| Natalie | Bowers | | | Genentech, San Francisco, CA, United States | Clinical Group / Task Force | |
| Edmond | Teng | | | Genentech, San Francisco, CA, United States | Clinical Group / Task Force | |
| Tim | Lu | | | Genentech, San Francisco, CA, United States | Clinical Group / Task Force | |
| Hubert | Chen | | | Genentech, San Francisco, CA, United States | Clinical Group / Task Force | |
| Jennifer | Schutzman | | | Genentech, San Francisco, CA, United States | Clinical Group / Task Force | |
| Erich | Strauss | | | Genentech, San Francisco, CA, United States | Clinical Group / Task Force | |

*Indicates required information. Only first name, last name, and suffix will appear in PubMed.

| First Name and Middle Initial(s)* | Last Name* | Suffix* | Academic Degrees | Institution and location (city, state/province, country) | Role or Contribution, eg, chair, principal investigator | Subgroup, eg, Steering Committee |
|---|---|---|---|---|---|---|
| Hao | Chen | | | Genentech, San Francisco, CA, United States | Clinical Group / Task Force | |
| David | Choy | | | Genentech, San Francisco, CA, United States | Clinical Group / Task Force | |
| Rion | Pendergrass | | | Genentech, San Francisco, CA, United States | Clinical Group / Task Force | |
| Brian | Yaspan | | | Genentech, San Francisco, CA, United States | Clinical Group / Task Force | |
| Cameron | Adams | | | Genentech, San Francisco, CA, United States | Clinical Group / Task Force | |
| Mark | McCarthy | | | Genentech, San Francisco, CA, United States | Clinical Group / Task Force | |
| Michael | Rothenberg | | | Genentech, San Francisco, CA, United States | Clinical Group / Task Force | |
| Rion | Pendergrass | | | Genentech, San Francisco, CA, United States | Clinical Group / Task Force | |
| Sergio | Dellepiane | | | Genentech, San Francisco, CA, United States | Clinical Group / Task Force | |
| Anubha | Mahajan | | | Genentech, San Francisco, CA, United States | Clinical Group / Task Force | |

Supplemental Online Content: Nonauthor Collaborators

*Indicates required information. Only first name, last name, and suffix will appear in PubMed.

| First Name and Middle Initial(s)* | Last Name* | Suffix* | Academic Degrees | Institution and location (city, state/province, country) | Role or Contribution, eg, chair, principal investigator | Subgroup, eg, Steering Committee |
|---|---|---|---|---|---|---|
| Michael | Holmes | | | Genentech, San Francisco, CA, United States | Clinical Group / Task Force | |
| Anubha | Mahajan | | | Genentech, San Francisco, CA, United States | Clinical Group / Task Force | |
| Diana | Chang | | | Genentech, San Francisco, CA, United States | Clinical Group / Task Force | |
| Tushar | Bhangale | | | Genentech, San Francisco, CA, United States | Clinical Group / Task Force | |
| Fanli | Xu | | | GlaxoSmithKline, Brentford, United Kingdom | Clinical Group / Task Force | |
| Laura | Addis | | | GlaxoSmithKline, Brentford, United Kingdom | Clinical Group / Task Force | |
| John | Eicher | | | GlaxoSmithKline, Brentford, United Kingdom | Clinical Group / Task Force | |
| Linda | McCarthy | | | GlaxoSmithKline, Brentford, United Kingdom | Clinical Group / Task Force | |
| Jorge | Esparza Gordillo | | | GlaxoSmithKline, Brentford, United Kingdom | Clinical Group / Task Force | |
| Joanna | Betts | | | GlaxoSmithKline, Brentford, United Kingdom | Clinical Group / Task Force | |

Supplemental Online Content: Nonauthor Collaborators

*Indicates required information. Only first name, last name, and suffix will appear in PubMed.

| First Name and Middle Initial(s)* | Last Name* | Suffix* | Academic Degrees | Institution and location (city, state/province, country) | Role or Contribution, eg, chair, principal investigator | Subgroup, eg, Steering Committee |
|---|---|---|---|---|---|---|
| Rajashree | Mishra | | | GlaxoSmithKline, Brentford, United Kingdom | Clinical Group / Task Force | |
| Audrey | Chu | | | GlaxoSmithKline, Brentford, United Kingdom | Clinical Group / Task Force | |
| Diptee | Kulkarni | | | GlaxoSmithKline, Brentford, United Kingdom | Clinical Group / Task Force | |
| Janet | Kumar | | | GlaxoSmithKline, Collegeville, PA, United States | Clinical Group / Task Force | |
| Charli | Harlow | | | GlaxoSmithKline, Collegeville, PA, United States | Clinical Group / Task Force | |
| Lea | Sarow-Blat | | | GlaxoSmithKline, Collegeville, PA, United States | Clinical Group / Task Force | |
| Diana | L.Cousminer | | | GlaxoSmithKline, Collegeville, PA, United States | Clinical Group / Task Force | |
| Jagtar | Nijjar | | | GlaxoSmithKline, Collegeville, PA, United States | Clinical Group / Task Force | |
| Jessica | Chao | | | GlaxoSmithKline, Collegeville, PA, United States | Clinical Group / Task Force | |
| Michal | Magid | | | GlaxoSmithKline, Collegeville, PA, United States | Clinical Group / Task Force | |

Supplemental Online Content: Nonauthor Collaborators

*Indicates required information. Only first name, last name, and suffix will appear in PubMed.

| First Name and Middle Initial(s)* | Last Name* | Suffix* | Academic Degrees | Institution and location (city, state/province, country) | Role or Contribution, eg, chair, principal investigator | Subgroup, eg, Steering Committee |
|---|---|---|---|---|---|---|
| Shashank | Jariwala | | | GlaxoSmithKline, Collegeville, PA, United States | Clinical Group / Task Force | |
| Chris | Floyd | | | GlaxoSmithKline, Collegeville, PA, United States | Clinical Group / Task Force | |
| Dan | Swerdlow | | | GlaxoSmithKline, Collegeville, PA, United States | Clinical Group / Task Force | |
| Erding | Hu | | | GlaxoSmithKline, Collegeville, PA, United States | Clinical Group / Task Force | |
| Prerak | Desai | | | GlaxoSmithKline, Collegeville, PA, United States | Clinical Group / Task Force | |
| Stephen | Haddad | | | GlaxoSmithKline, Collegeville, PA, United States | Clinical Group / Task Force | |
| Damien | Croteau-Chonka | | | GlaxoSmithKline, Collegeville, PA, United States | Clinical Group / Task Force | |
| Billy | Fahy | | | GlaxoSmithKline, Collegeville, PA, United States | Clinical Group / Task Force | |
| Paola | Bronson | | | GlaxoSmithKline, Collegeville, PA, United States | Clinical Group / Task Force | |
| Kirsi | Auro | | | GlaxoSmithKline, Espoo, Finland | Clinical Group / Task Force | |

Supplemental Online Content: Nonauthor Collaborators

*Indicates required information. Only first name, last name, and suffix will appear in PubMed.

| First Name and Middle Initial(s)* | Last Name* | Suffix* | Academic Degrees | Institution and location (city, state/province, country) | Role or Contribution, eg, chair, principal investigator | Subgroup, eg, Steering Committee |
|---|---|---|---|---|---|---|
| David | Pulford | | | GlaxoSmithKline, Stevenage, United Kingdom | Clinical Group / Task Force | |
| Sauli | Vuoti | | | Janssen-Cilag Oy, Espoo, Finland | Clinical Group / Task Force | |
| Dermot | Reilly | | | Johnson & Johnson Innovative Medicine, Boston, MA, United States | Clinical Group / Task Force | |
| Karen | He | | | Johnson & Johnson Innovative Medicine, Spring House, PA, United States | Clinical Group / Task Force | |
| Ekaterina | Khramtsova | | | Johnson & Johnson Innovative Medicine, Spring House, PA, United States | Clinical Group / Task Force | |
| Amy | Hart | | | Johnson & Johnson Innovative Medicine, Spring House, PA, United States | Clinical Group / Task Force | |
| Meijian | Guan | | | Johnson & Johnson Innovative Medicine, Spring House, PA, United States | Clinical Group / Task Force | |
| Alessandro | Porello | | | Johnson & Johnson Innovative Medicine, Spring House, PA, United States | Clinical Group / Task Force | |

Supplemental Online Content: Nonauthor Collaborators

*Indicates required information. Only first name, last name, and suffix will appear in PubMed.

| First Name and Middle Initial(s)* | Last Name* | Suffix* | Academic Degrees | Institution and location (city, state/province, country) | Role or Contribution, eg, chair, principal investigator | Subgroup, eg, Steering Committee |
|---|---|---|---|---|---|---|
| P. | Dunnmon | | | Johnson & Johnson Innovative Medicine, Spring House, PA, United States | Clinical Group / Task Force | |
| Sara | Gale | | | Johnson & Johnson Innovative Medicine, Spring House, PA, United States | Clinical Group / Task Force | |
| Brice | Keyes | | | Johnson & Johnson Innovative Medicine, Spring House, PA, United States | Clinical Group / Task Force | |
| John | Kwon | | | Johnson & Johnson Innovative Medicine, Spring House, PA, United States | Clinical Group / Task Force | |
| Jonathan | Sherlock | | | Johnson & Johnson Innovative Medicine, Spring House, PA, United States | Clinical Group / Task Force | |
| Matt | Loza | | | Johnson & Johnson Innovative Medicine, Spring House, PA, United States | Clinical Group / Task Force | |
| Chris | Whelan | | | Johnson & Johnson Innovative Medicine, Spring House, PA, United States | Clinical Group / Task Force | |

Supplemental Online Content: Nonauthor Collaborators

*Indicates required information. Only first name, last name, and suffix will appear in PubMed.

| First Name and Middle Initial(s)* | Last Name* | Suffix* | Academic Degrees | Institution and location (city, state/province, country) | Role or Contribution, eg, chair, principal investigator | Subgroup, eg, Steering Committee |
|---|---|---|---|---|---|---|
| W | Galpern | | | Johnson & Johnson Innovative Medicine, Spring House, PA, United States | Clinical Group / Task Force | |
| Yanfei | Zhang | | | Johnson & Johnson Innovative Medicine, Spring House, PA, United States | Clinical Group / Task Force | |
| Mona | Selej | | | Johnson & Johnson Innovative Medicine, Spring House, PA, United States | Clinical Group / Task Force | |
| Abolfazl | Doostparast Torshizi | | | Johnson & Johnson Innovative Medicine, Spring House, PA, United States | Clinical Group / Task Force | |
| Qingqin S | Li | | | Johnson & Johnson Innovative Medicine, Titusville, NJ, United States | Clinical Group / Task Force | |
| Sahar | Mozzafari | | | Maze Therapeutics, San Francisco, CA, United States | Clinical Group / Task Force | |
| Christopher | Deboever | | | Maze Therapeutics, San Francisco, CA, United States | Clinical Group / Task Force | |
| Jason | Miller | | | Merck, Kenilworth, NJ, United States | Clinical Group / Task Force | |
| Fabiana | Farias | | | Merck, Kenilworth, NJ, United States | Clinical Group / Task Force | |

Supplemental Online Content: Nonauthor Collaborators

*Indicates required information. Only first name, last name, and suffix will appear in PubMed.

| First Name and Middle Initial(s)* | Last Name* | Suffix* | Academic Degrees | Institution and location (city, state/province, country) | Role or Contribution, eg, chair, principal investigator | Subgroup, eg, Steering Committee |
|---|---|---|---|---|---|---|
| Andrey | Loboda | | | Merck, Kenilworth, NJ, United States | Clinical Group / Task Force | |
| Jorge | Del-aguila | | | Merck, Kenilworth, NJ, United States | Clinical Group / Task Force | |
| Elisabeth | Vollmann | | | Merck, Kenilworth, NJ, United States | Clinical Group / Task Force | |
| Jozsef | Karman | | | Merck, Kenilworth, NJ, United States | Clinical Group / Task Force | |
| Julie | Fiore | | | Merck, Kenilworth, NJ, United States | Clinical Group / Task Force | |
| Rajesh | Kamath | | | Merck, Kenilworth, NJ, United States | Clinical Group / Task Force | |
| Andrei | Popescu | | | Merck, Kenilworth, NJ, United States | Clinical Group / Task Force | |
| Delphine | Fagegaltier | | | Merck, Kenilworth, NJ, United States | Clinical Group / Task Force | |
| Travis | Barr | | | Merck, Kenilworth, NJ, United States | Clinical Group / Task Force | |
| Aristide | Merola | | | Merck, Kenilworth, NJ, United States | Clinical Group / Task Force | |
| Oliver | Freeman | | | Merck, Kenilworth, NJ, United States | Clinical Group / Task Force | |
| Simonne | Longerich | | | Merck, Kenilworth, NJ, United States | Clinical Group / Task Force | |
| Enrico | Ferrero | | | Novartis Institutes for BioMedical Research, Cambridge, MA, United States | Clinical Group / Task Force | |

Supplemental Online Content: Nonauthor Collaborators

*Indicates required information. Only first name, last name, and suffix will appear in PubMed.

| First Name and Middle Initial(s)* | Last Name* | Suffix* | Academic Degrees | Institution and location (city, state/province, country) | Role or Contribution, eg, chair, principal investigator | Subgroup, eg, Steering Committee |
|---|---|---|---|---|---|---|
| Nikos | Patsopoulos | | | Novartis Institutes for BioMedical Research, Cambridge, MA, United States | Clinical Group / Task Force | |
| Nancy | Finkel | | | Novartis Institutes for BioMedical Research, Cambridge, MA, United States | Clinical Group / Task Force | |
| Sabina | Pfister | | | Novartis Institutes for BioMedical Research, Cambridge, MA, United States | Clinical Group / Task Force | |
| Shola | Richards | | | Novartis Institutes for BioMedical Research, Cambridge, MA, United States | Clinical Group / Task Force | |
| Katherine | Mccauley | | | Novartis Institutes for BioMedical Research, Cambridge, MA, United States | Clinical Group / Task Force | |
| Xiaobo | Xia | | | Novartis Institutes for BioMedical Research, Cambridge, MA, United States | Clinical Group / Task Force | |
| Mike | Mendelson | | | Novartis Institutes for BioMedical Research, Cambridge, MA, United States | Clinical Group / Task Force | |
| Majd | Mouded | | | Novartis, Basel, Switzerland | Clinical Group / Task Force | |

Supplemental Online Content: Nonauthor Collaborators

*Indicates required information. Only first name, last name, and suffix will appear in PubMed.

| First Name and Middle Initial(s)* | Last Name* | Suffix* | Academic Degrees | Institution and location (city, state/province, country) | Role or Contribution, eg, chair, principal investigator | Subgroup, eg, Steering Committee |
|---|---|---|---|---|---|---|
| Debby | Ngo | | | Novartis, Basel, Switzerland | Clinical Group / Task Force | |
| Kirsi | Kalpala | | | Pfizer, New York, NY, United States | Clinical Group / Task Force | |
| Melissa | Miller | | | Pfizer, New York, NY, United States | Clinical Group / Task Force | |
| Nan | Bing | | | Pfizer, New York, NY, United States | Clinical Group / Task Force | |
| Jaakko | Parkkinen | | | Pfizer, New York, NY, United States | Clinical Group / Task Force | |
| Heli | Lehtonen | | | Pfizer, New York, NY, United States | Clinical Group / Task Force | |
| Stefan | McDonough | | | Pfizer, New York, NY, United States | Clinical Group / Task Force | |
| Ying | Wu | | | Pfizer, New York, NY, United States | Clinical Group / Task Force | |
| Erin | Macdonald-Dunlop | | | Pfizer, New York, NY, United States | Clinical Group / Task Force | |
| Jessica | Chung | | | Pfizer, New York, NY, United States | Clinical Group / Task Force | |
| Michael | McLean | | | Pfizer, New York, NY, United States | Clinical Group / Task Force | |
| Joshua | Chiou | | | Pfizer, New York, NY, United States | Clinical Group / Task Force | |
| Hye | In Kim | | | Pfizer, New York, NY, United States | Clinical Group / Task Force | |
| Sivakumar | Pitchumani | | | Pfizer, New York, NY, United States | Clinical Group / Task Force | |
| Sumedha | Jassal | | | Pfizer, New York, NY, United States | Clinical Group / Task Force | |

Supplemental Online Content: Nonauthor Collaborators

*Indicates required information. Only first name, last name, and suffix will appear in PubMed.

| First Name and Middle Initial(s)* | Last Name* | Suffix* | Academic Degrees | Institution and location (city, state/province, country) | Role or Contribution, eg, chair, principal investigator | Subgroup, eg, Steering Committee |
|---|---|---|---|---|---|---|
| Madhurima | Saxena | | | Pfizer, New York, NY, United States | Clinical Group / Task Force | |
| Catherine | O'Riordan | | | Translational Sciences, Sanofi R&D, Framingham, MA, USA | Clinical Group / Task Force | |
| Samuel | Lessard | | | Translational Sciences, Sanofi R&D, Framingham, MA, USA | Clinical Group / Task Force | |
| Suzanne | Jacobs | | | Translational Sciences, Sanofi R&D, Framingham, MA, USA | Clinical Group / Task Force | |
| Hamid | Mattoo | | | Translational Sciences, Sanofi R&D, Framingham, MA, USA | Clinical Group / Task Force | |
| David | Habiel | | | Translational Sciences, Sanofi R&D, Framingham, MA, USA | Clinical Group / Task Force | |
| Guanling | Huan | | | Translational Sciences, Sanofi R&D, Framingham, MA, USA | Clinical Group / Task Force | |
| Lila | Kallio | | | Auria Biobank / University of Turku / Wellbeing Services County of Southwest Finland, Turku, Finland | Biobank directors | |
| Tiina | Wahlfors | | | THL Biobank / Finnish Institute for Health and Welfare (THL), Helsinki, Finland | Biobank directors | |

Supplemental Online Content: Nonauthor Collaborators

*Indicates required information. Only first name, last name, and suffix will appear in PubMed.

| First Name and Middle Initial(s)* | Last Name* | Suffix* | Academic Degrees | Institution and location (city, state/province, country) | Role or Contribution, eg, chair, principal investigator | Subgroup, eg, Steering Committee |
|---|---|---|---|---|---|---|
| Jukka | Partanen | | | Finnish Red Cross Blood Service / Finnish Hematology Registry and Clinical Biobank, Helsinki, Finland | Biobank directors | |
| Eero | Punkka | | | Helsinki Biobank / Helsinki University and Hospital District of Helsinki and Uusimaa, Helsinki | Biobank directors | |
| Raisa | Serpi | | | Northern Finland Biobank Borealis / University of Oulu / Wellbeing services county of North Ostrobothnia, Oulu, Finland | Biobank directors | |
| Sanna | Siltanen | | | Finnish Clinical Biobank Tampere / University of Tampere / Wellbeing Services County of Pirkanmaa, Tampere, Finland | Biobank directors | |
| Veli-Matti | Kosma | | | Biobank of Eastern Finland / University of Eastern Finland / Wellbeing services county of North Savo, Kuopio, Finland | Biobank directors | |

Supplemental Online Content: Nonauthor Collaborators

*Indicates required information. Only first name, last name, and suffix will appear in PubMed.

| First Name and Middle Initial(s)* | Last Name* | Suffix* | Academic Degrees | Institution and location (city, state/province, country) | Role or Contribution, eg, chair, principal investigator | Subgroup, eg, Steering Committee |
|---|---|---|---|---|---|---|
| Tiina | Jokela | | | Central Finland Biobank / University of Jyväskylä / Wellbeing Services County of Central Finland, Jyväskylä, Finland | Biobank directors | |
| Anu | Jalanko | | | Institute for Molecular Medicine Finland (FIMM), HiLIFE, University of Helsinki, Helsinki, Finland | FinnGen Teams | Administration |
| Risto | Kajanne | | | Institute for Molecular Medicine Finland (FIMM), HiLIFE, University of Helsinki, Helsinki, Finland | FinnGen Teams | Administration |
| Mervi | Aavikko | | | Institute for Molecular Medicine Finland (FIMM), HiLIFE, University of Helsinki, Helsinki, Finland | FinnGen Teams | Administration |
| Helen | Cooper | | | Institute for Molecular Medicine Finland (FIMM), HiLIFE, University of Helsinki, Helsinki, Finland | FinnGen Teams | Administration |
| Denise | Öller | | | Institute for Molecular Medicine Finland (FIMM), HiLIFE, University of Helsinki, Helsinki, Finland | FinnGen Teams | Administration |

Supplemental Online Content: Nonauthor Collaborators

*Indicates required information. Only first name, last name, and suffix will appear in PubMed.

| First Name and Middle Initial(s)* | Last Name* | Suffix* | Academic Degrees | Institution and location (city, state/province, country) | Role or Contribution, eg, chair, principal investigator | Subgroup, eg, Steering Committee |
|---|---|---|---|---|---|---|
| Tarja | Laitinen | | | Institute for Molecular Medicine Finland (FIMM), HiLIFE, University of Helsinki, Helsinki, Finland | FinnGen Teams | Administration |
| Sofia | Kuitunen | | | University of Helsinki, Helsinki, Finland | FinnGen Teams | Administration |
| Auli | Toivola | | | Institute for Molecular Medicine Finland (FIMM), HiLIFE, University of Helsinki, Helsinki, Finland | FinnGen Teams | Sample and data logistics |
| Rodos | Rodosthenous | | | Institute for Molecular Medicine Finland (FIMM), HiLIFE, University of Helsinki, Helsinki, Finland | FinnGen Teams | Sample and data logistics |
| Mitja | Kurki | | | Institute for Molecular Medicine Finland (FIMM), HiLIFE, University of Helsinki, Helsinki, Finland; Broad Institute, Cambridge, MA, United States | FinnGen Teams | Analysis |
| Juha | Karjalainen | | | Institute for Molecular Medicine Finland (FIMM), HiLIFE, University of Helsinki, Helsinki, Finland | FinnGen Teams | Analysis |

Supplemental Online Content: Nonauthor Collaborators

*Indicates required information. Only first name, last name, and suffix will appear in PubMed.

| First Name and Middle Initial(s)* | Last Name* | Suffix* | Academic Degrees | Institution and location (city, state/province, country) | Role or Contribution, eg, chair, principal investigator | Subgroup, eg, Steering Committee |
|---|---|---|---|---|---|---|
| Pietro | Della Briotta Parolo | | | Institute for Molecular Medicine Finland (FIMM), HiLIFE, University of Helsinki, Helsinki, Finland | FinnGen Teams | Analysis |
| Arto | Lehisto | | | Institute for Molecular Medicine Finland (FIMM), HiLIFE, University of Helsinki, Helsinki, Finland | FinnGen Teams | Analysis |
| Juha | Mehtonen | | | Institute for Molecular Medicine Finland (FIMM), HiLIFE, University of Helsinki, Helsinki, Finland | FinnGen Teams | Analysis |
| Reza | Jabal | | | Institute for Molecular Medicine Finland (FIMM), HiLIFE, University of Helsinki, Helsinki, Finland; Broad Institute, Cambridge, MA, United States | FinnGen Teams | Analysis |
| Mutaamba | Maasha | | | Institute for Molecular Medicine Finland (FIMM), HiLIFE, University of Helsinki, Helsinki, Finland; Broad Institute, Cambridge, MA, United States | FinnGen Teams | Analysis |

Supplemental Online Content: Nonauthor Collaborators

*Indicates required information. Only first name, last name, and suffix will appear in PubMed.

| First Name and Middle Initial(s)* | Last Name* | Suffix* | Academic Degrees | Institution and location (city, state/province, country) | Role or Contribution, eg, chair, principal investigator | Subgroup, eg, Steering Committee |
|---|---|---|---|---|---|---|
| Sanni | Ruotsalainen | | | Institute for Molecular Medicine Finland (FIMM), HiLIFE, University of Helsinki, Helsinki, Finland | FinnGen Teams | Analysis |
| Samuel | Jones | | | Institute for Molecular Medicine Finland (FIMM), HiLIFE, University of Helsinki, Helsinki, Finland | FinnGen Teams | Analysis |
| Raymond | Walters | | | Institute for Molecular Medicine Finland (FIMM), HiLIFE, University of Helsinki, Helsinki, Finland; Broad Institute, Cambridge, MA, United States | FinnGen Teams | Analysis |
| Paavo | Häppölä | | | Institute for Molecular Medicine Finland (FIMM), HiLIFE, University of Helsinki, Helsinki, Finland | FinnGen Teams | Analysis |
| L. Elisa | Lahtela | | | Institute for Molecular Medicine Finland (FIMM), HiLIFE, University of Helsinki, Helsinki, Finland | FinnGen Teams | Disease Task Forces |

Supplemental Online Content: Nonauthor Collaborators

*Indicates required information. Only first name, last name, and suffix will appear in PubMed.

| First Name and Middle Initial(s)* | Last Name* | Suffix* | Academic Degrees | Institution and location (city, state/province, country) | Role or Contribution, eg, chair, principal investigator | Subgroup, eg, Steering Committee |
|---|---|---|---|---|---|---|
| Johanna | Paltta | | | Institute for Molecular Medicine Finland (FIMM), HiLIFE, University of Helsinki, Helsinki, Finland; University of Turku, Turku, Finland | FinnGen Teams | Disease Task Forces |
| Juulia | Partanen | | | Institute for Molecular Medicine Finland, HiLIFE, University of Helsinki, Finland | FinnGen Teams | Disease Task Forces |
| Mari | Kaunisto | | | Institute for Molecular Medicine Finland (FIMM), HiLIFE, University of Helsinki, Helsinki, Finland | FinnGen Teams | Communication |
| Elina | Kilpeläinen | | | Institute for Molecular Medicine Finland (FIMM), HiLIFE, University of Helsinki, Helsinki, Finland | FinnGen Teams | Sandbox & Cloud Services |
| Tianduanyi | Wang | | | Institute for Molecular Medicine Finland (FIMM), HiLIFE, University of Helsinki, Helsinki, Finland | FinnGen Teams | Sandbox & Cloud Services |
| Timo P. | Sipilä | | | Institute for Molecular Medicine Finland (FIMM), HiLIFE, University of Helsinki, Helsinki, Finland | FinnGen Teams | Sandbox & Cloud Services |

Supplemental Online Content: Nonauthor Collaborators

*Indicates required information. Only first name, last name, and suffix will appear in PubMed.

| First Name and Middle Initial(s)* | Last Name* | Suffix* | Academic Degrees | Institution and location (city, state/province, country) | Role or Contribution, eg, chair, principal investigator | Subgroup, eg, Steering Committee |
|---|---|---|---|---|---|---|
| Oluwaseun Alexander | Dada | | | Institute for Molecular Medicine Finland (FIMM), HiLIFE, University of Helsinki, Helsinki, Finland | FinnGen Teams | Sandbox & Cloud Services |
| Awaisa | Ghazal | | | Institute for Molecular Medicine Finland (FIMM), HiLIFE, University of Helsinki, Helsinki, Finland | FinnGen Teams | Sandbox & Cloud Services |
| Rigbe | Weldatsadik | | | Institute for Molecular Medicine Finland (FIMM), HiLIFE, University of Helsinki, Helsinki, Finland | FinnGen Teams | Sandbox & Cloud Services |
| Jaska | Uimonen | | | Institute for Molecular Medicine Finland (FIMM), HiLIFE, University of Helsinki, Helsinki, Finland | FinnGen Teams | Sandbox & Cloud Services |
| Kati | Donner | | | Institute for Molecular Medicine Finland (FIMM), HiLIFE, University of Helsinki, Helsinki, Finland | FinnGen Teams | Genotyping |
| Anu | Loukola | | | Helsinki Biobank / Helsinki University and Hospital District of Helsinki and Uusimaa, Helsinki | FinnGen Teams | Sample Collection Coordination |

Supplemental Online Content: Nonauthor Collaborators

*Indicates required information. Only first name, last name, and suffix will appear in PubMed.

| First Name and Middle Initial(s)* | Last Name* | Suffix* | Academic Degrees | Institution and location (city, state/province, country) | Role or Contribution, eg, chair, principal investigator | Subgroup, eg, Steering Committee |
|---|---|---|---|---|---|---|
| Päivi | Laiho | | | THL Biobank / Finnish Institute for Health and Welfare (THL), Helsinki, Finland | FinnGen Teams | Sample Logistics |
| Susanna | Lemmelä | | | Institute for Molecular Medicine Finland (FIMM), HiLIFE, University of Helsinki, Helsinki, Finland | FinnGen Teams | Registry Data Operations |
| Teemu | Paajanen | | | THL Biobank / Finnish Institute for Health and Welfare (THL), Helsinki, Finland | FinnGen Teams | Registry Data Operations |
| Arto | Pietilä | | | THL Biobank / Finnish Institute for Health and Welfare (THL), Helsinki, Finland | FinnGen Teams | Registry Data Operations |
| Aki | Havulinna | | | THL Biobank / Finnish Institute for Health and Welfare (THL), Helsinki, Finland | FinnGen Teams | Registry Data Operations |
| Mary Pat | Reeve | | | Institute for Molecular Medicine Finland (FIMM), HiLIFE, University of Helsinki, Helsinki, Finland; Broad Institute, Cambridge, MA, United States | FinnGen Teams | Phenotype team |

Supplemental Online Content: Nonauthor Collaborators

*Indicates required information. Only first name, last name, and suffix will appear in PubMed.

| First Name and Middle Initial(s)* | Last Name* | Suffix* | Academic Degrees | Institution and location (city, state/province, country) | Role or Contribution, eg, chair, principal investigator | Subgroup, eg, Steering Committee |
|---|---|---|---|---|---|---|
| Shanmukha Sampath | Padmanabhuni | | | Institute for Molecular Medicine Finland (FIMM), HiLIFE, University of Helsinki, Helsinki, Finland | FinnGen Teams | Phenotype team |
| Harri | Siirtola | | | University of Tampere, Tampere, Finland | FinnGen Teams | Phenotype team |
| Javier | Gracia-Tabuenca | | | University of Tampere, Tampere, Finland | FinnGen Teams | Phenotype team |
| Marika | Kaakinen | | | Institute for Molecular Medicine Finland (FIMM), HiLIFE, University of Helsinki, Helsinki, Finland | FinnGen Teams | Phenotype team |
| Shuang | Luo | | | Institute for Molecular Medicine Finland (FIMM), HiLIFE, University of Helsinki, Helsinki, Finland | FinnGen Teams | Phenotype team |
| Vincent | Llorens | | | Institute for Molecular Medicine Finland (FIMM), HiLIFE, University of Helsinki, Helsinki, Finland | FinnGen Teams | Phenotype team |
| Dawit | Yohannes | | | Institute for Molecular Medicine Finland (FIMM), HiLIFE, University of Helsinki, Helsinki, Finland | FinnGen Teams | Phenotype team |

Supplemental Online Content: Nonauthor Collaborators

*Indicates required information. Only first name, last name, and suffix will appear in PubMed.

| First Name and Middle Initial(s)* | Last Name* | Suffix* | Academic Degrees | Institution and location (city, state/province, country) | Role or Contribution, eg, chair, principal investigator | Subgroup, eg, Steering Committee |
|---|---|---|---|---|---|---|
| Iina | Laak | | | Institute for Molecular Medicine Finland (FIMM), HiLIFE, University of Helsinki, Helsinki, Finland | FinnGen Teams | Data protection officer |
| Mervi | Ahlroth | | | Finnish Biobank Cooperative - FINBB | FinnGen Teams | FINBB - Finnish biobank cooperative |
| Johanna | Mäkelä | | | Finnish Biobank Cooperative - FINBB | FinnGen Teams | FINBB - Finnish biobank cooperative |
| Pauli | Wihuri | | | Finnish Biobank Cooperative - FINBB | FinnGen Teams | FINBB - Finnish biobank cooperative |
| Tom | Southerington | | | Finnish Biobank Cooperative - FINBB | FinnGen Teams | FINBB - Finnish biobank cooperative |
| Meri | Lähteenmäki | | | Finnish Biobank Cooperative - FINBB | FinnGen Teams | FINBB - Finnish biobank cooperative |
