# Supplementary material for: XXYLT1 and Mendelian Retinal Dystrophy
Source: JAMA Ophthalmol. 2026 Jul 30:e262795. Online ahead of print. doi: 10.1001/jamaophthalmol.2026.2795 (PMC13425242; doi:10.1001/jamaophthalmol.2026.2795)
Supplement: Supplement 3. — Data sharing statement [file jamaophthalmol-e262795-s003.pdf]

Data Sharing Statement

Kraatari-Tiri. XXYLT1 and Mendelian Retinal Dystrophy. *JAMA Ophthalmol*. Published July 30, 2026. doi:10.1001/jamaophthalmol.2026.2795

Data
Data available: No

Additional Information
Explanation for why data not available: Based on National and European regulations (GDPR), access to individual-level sensitive health data must be approved by national authorities. Finnish biobank data can be accessed through the Fingenious services (https://site.fingenious.fi/en/) managed by FINBB (https://finbb.fi/). Finnish Health register data can be applied from Findata (https://findata.fi/en/data/). Information on accessing FinnGen data can be found at https://www.finngen.fi/en/access_results/.
